# Lithium isotope evidence for enhanced continental weathering triggering the late Paleozoic greenhouse-to-icehouse climate transition

**DOI:** 10.1093/nsr/nwag168

**Published:** 2026-03-17

**Authors:** Feifei Zhang, Guang-Yi Wei, Pierre Maffre, Ziheng Li, Jianlin Zhou, Alexandre Pohl, Yi-Bo Lin, Maya Elrick, Keyi Cheng, Philip A E Pogge von Strandmann, Shu-zhong Shen

**Affiliations:** State Key Laboratory of Critical Earth Material Cycling and Mineral Deposits, School of Earth Sciences and Engineering, and Frontiers Science Center for Critical Earth Material Cycling, Nanjing University, Nanjing 210023, China; State Key Laboratory of Critical Earth Material Cycling and Mineral Deposits, School of Earth Sciences and Engineering, and Frontiers Science Center for Critical Earth Material Cycling, Nanjing University, Nanjing 210023, China; CNRS, IRD, INRAE, CEREGE, Aix-Marseille University, Aix-en-Provence 13007, France; State Key Laboratory of Geomicrobiology and Environmental Changes, China University of Geosciences, Wuhan 430074, China; State Key Laboratory of Critical Earth Material Cycling and Mineral Deposits, School of Earth Sciences and Engineering, and Frontiers Science Center for Critical Earth Material Cycling, Nanjing University, Nanjing 210023, China; CNRS, Biogéosciences UMR 6282, Université Bourgogne Europe, Dijon 21000, France; State Key Laboratory of Critical Earth Material Cycling and Mineral Deposits, School of Earth Sciences and Engineering, and Frontiers Science Center for Critical Earth Material Cycling, Nanjing University, Nanjing 210023, China; Department of Earth & Planetary Sciences, University of New Mexico, Albuquerque, NM 87131, USA; School of Earth and Ocean Sciences, University of Victoria, Victoria V8P5C2, Canada; MIGHTY (Mainz Isotope and Geochemistry Centre), Institute of Geosciences, Johannes Gutenberg University, Mainz 55122, Germany; State Key Laboratory of Critical Earth Material Cycling and Mineral Deposits, School of Earth Sciences and Engineering, and Frontiers Science Center for Critical Earth Material Cycling, Nanjing University, Nanjing 210023, China

**Keywords:** lithium isotope, continental weathering, Late Paleozoic Ice Age, carbon cycle perturbation, deep-time Earth system modeling

## Abstract

The causes of the transition from a greenhouse to an icehouse climate during the Early Mississippian (∼359–347 Ma), Carboniferous, marking the possible onset of the Late Paleozoic Ice Age (LPIA), remain an outstanding puzzle in Earth’s climate history. This transition coincides with the mid-Tournaisian carbon isotope excursion (TICE). It is hypothesized that intensified continental silicate weathering during this period sequestered CO₂, boosted nutrients, and increased marine productivity, causing the positive TICE. However, direct evidence for enhanced continental weathering has been lacking. We present lithium isotope (δ⁷Li) data from two carbonate sections showing a ∼12‰ decline during TICE. Integrating δ⁷Li with carbon cycle models (GEOCLIM and COPSE) suggests silicate chemical weathering rates increased ∼30% with declines in weathering intensity during the TICE event, likely due to uplift or vegetation, ultimately reducing atmospheric *p*CO₂ by ∼1000 ppmv. This provides evidence linking enhanced chemical weathering, CO₂ drawdown, and the onset of the LPIA, shedding light on a key aspect of late Paleozoic climate change.

## Teaser

Carbonate lithium isotopes and biogeochemical models indicate significant increases in global continental chemical weathering rates, driving substantial declines in atmospheric CO_2_ that initiated the onset of the Late Paleozoic Ice Age.

## INTRODUCTION

The shift from a middle Paleozoic greenhouse climate to a late Paleozoic icehouse—marking the onset of the Late Paleozoic Ice Age (LPIA)—represents one of the most profound climatic transitions in Earth’s history. While the exact timing of the onset of LPIA is still under debate, available evidence suggests that this climatic transition probably began during the Early Mississippian, Carboniferous, coinciding with one of the most prominent positive carbonate carbon isotope excursions (δ^13^C_carb_) documented in the geological record [1–3]. This event, referred to as the mid-Tournaisian carbon isotope excursion (TICE), is characterized by δ^13^C_carb_ values reaching up to +7‰ and persisted for ∼4 Myr, with records documented across multiple regions spanning different continents [4–7]. Meanwhile, conodont apatite and brachiopod shell oxygen isotope data indicates a profound cooling of 6–8°C during the TICE interval [2,8,9], likely representing the initiation of LPIA.

**Figure 1.**
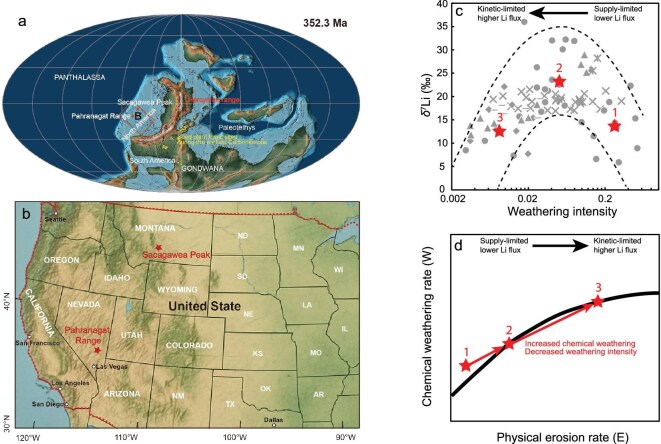
The geological localities of two study sections, and relationships between Li isotope and weathering intensity based on modern river systems. (a) Early Mississippian paleogeography of North America, modified from Scotese and Wright [67]. The stars indicate the locations of the study areas in present-day. (b) A US map showing the modern localities of SP and PR. The distributions of seed plant fossil sites in (a) were modified from Chen *et al.* [8]. (c) Relationships between riverine dissolved δ^7^Li and weathering intensity, showing the ‘boomerang’ shape based on data from modern river systems [30]. (d) The schematic diagram of correlations between chemical weathering and physical erosion rates [25,26]. The stars in (c) and (d) denote the relationships among chemical weathering rate, physical erosion rate, and weathering intensity, exhibiting the trend of increasing physical erosion and chemical weathering rates but decreasing weathering intensity.

Explaining the mechanisms driving this climate shift to the Carboniferous glaciation remains an outstanding puzzle in geological and paleo-climatic sciences. In addition to long-term changes in volcanic degassing from the Devonian to the Carboniferous [10], it is widely hypothesized that chemical weathering of silicate rocks and/or burial of marine organic carbon played vital roles in driving the decrease in atmospheric CO_2_ concentrations, by sequestering atmospheric carbon in marine sediments via inorganic or reduced organic forms, as carbonates or black shales [4,8,11]. For instance, enhanced physical erosion associated with the development of Variscan orogeny in the equatorial area, and increased global continental weatherability due to the northward movement of Pangea has been notably proposed as a trigger for the stimulated chemical weathering and CO_2_ drawdown accompanying the LPIA [12,13] (Fig. 1a). Meanwhile, elevated continental silicate weathering can enhance the delivery of nutrients (for example, phosphorus and silicon) to the ocean, stimulating marine primary productivity and promoting export production and sequestration of carbon to the deep ocean. Indeed, isotopic studies of nitrogen, zinc, and barium indicate a substantial increase in marine primary productivity during the Early Mississippian [14–16]. While the prevailing model attributes the greenhouse-to-icehouse transition during the late Paleozoic primarily to CO₂ drawdown via enhanced silicate weathering and the subsequent weathering-driven productivity increase and organic carbon burial [8,14,15], direct evidence for increased silicate weathering during the Early Mississippian and its triggers remains unclear. Variations in radiogenic strontium isotope ratios (^87^Sr/^86^Sr)—which decline during the TICE—have been interpreted as indicative of intensified basalt weathering [8]. However, the interpretation of these ratios is complicated because they reflect mixed contributions from carbonate and silicate weathering as well as influence from hydrothermal activity, which have different impacts on atmospheric CO_2_ and climate [17,18]. Hence, the traditional proxy of radiogenic strontium isotopes may not provide direct constraints on global continental silicate weathering that ultimately regulate atmospheric CO_2_ level.

Lithium isotopes (δ^7^Li) in marine carbonates are a highly promising proxy for tracking continental silicate weathering, as δ^7^Li is generally not fractionated by biological processes [19] nor influenced by carbonate weathering during continental chemical weathering on Earth’s surface [20]. The δ^7^Li of primary silicate rocks—such as the upper continental crust (δ^7^Li ≈ +0.6‰ ± 0.6‰) and basalts (δ^7^Li ≈ +3‰–+5‰)—shows relatively limited variability [21]. During silicate weathering, the lighter ^6^Li isotope is preferentially incorporated into secondary clay minerals, leaving the dissolved lithium in rivers enriched in ^7^Li [22–24]. This isotopic fractionation results in riverine δ^7^Li values ranging from +2‰ to +43‰, with higher δ^7^Li values indicating medium weathering intensity (*W*/*D*, defined as the ratio of chemical weathering rate to total denudation rate, and total denudation rate equals to the sum of chemical weathering and physical erosion rates) and involving extensive clay formation. In contrast, lower riverine δ^7^Li signatures reflect either low or high weathering intensity with minimal clay precipitation [20] (Fig. 1c). It should be noted that chemical weathering typically exhibits a non-linear correlation to physical erosion, reflecting transitions from supply-limited conditions (low physical erosion but high weathering intensity) to kinetic-limited conditions (high physical erosion but low weathering intensity) [25,26] (Fig. 1d). The global seawater δ^7^Li budget is controlled by inputs—mainly riverine fluxes that are closely related to weathering intensities and high-temperature hydrothermal fluids—and outputs, such as sequestration into authigenic clays and altered oceanic crust [27–29]. Hydrothermal isotope ratios are relatively constant δ⁷Li ≈ +8‰, whereas riverine inputs vary with silicate weathering intensity [30]. The condition of low weathering intensity corresponds to high riverine Li flux and low δ^7^Li, ultimately causing rapid declines in seawater δ⁷Li, while intermediate weathering intensity results in enhanced secondary clay formation and elevates seawater δ^7^Li [20,28,29]. Additionally, the condition of high weathering intensity is characterized by low riverine Li flux and low δ^7^Li, and cannot notably affect δ^7^Li under relatively short time scales [28,29]. Taken together, temporal changes in seawater δ^7^Li can directly reflect the balance between primary silicate dissolution and secondary clay formation, providing a sensitive and reliable record of continental silicate weathering intensity and their feedbacks on climate. This relationship has been documented during critical climatic events such as the Precambrian–Cambrian transition [31,32], the Late Ordovician [17], the Permian–Triassic mass extinction [33,34], Cretaceous oceanic anoxic event [35], and the Paleocene–Eocene thermal maximum [18,36].

In this study, we present high-resolution δ^7^Li data obtained from two widely distributed shallow-water carbonate sections spanning the TICE—the Pahranaghat range (PR) section in southern Nevada (number of samples = 37) and the Sacagawea Peak (SP) section in southwest Montana (number of samples = 39), USA, representing carbonate deposition in different basins during the Early Carboniferous (Fig. 1a, b). Then, we use these new constraints to guide long-term carbon cycle models to investigate the drivers of the reported δ^7^Li trends and quantify the changes in silicate weathering and resulting Earth system changes during the TICE. By integrating carbonate δ^7^Li and δ^13^C data with process-based models, our work provides direct constraints on how silicate weathering contributed to the early phases of the LPIA and the broader climate evolution during Earth’s critical transition into the penultimate glaciation.

## RESULTS

**Figure 2.**
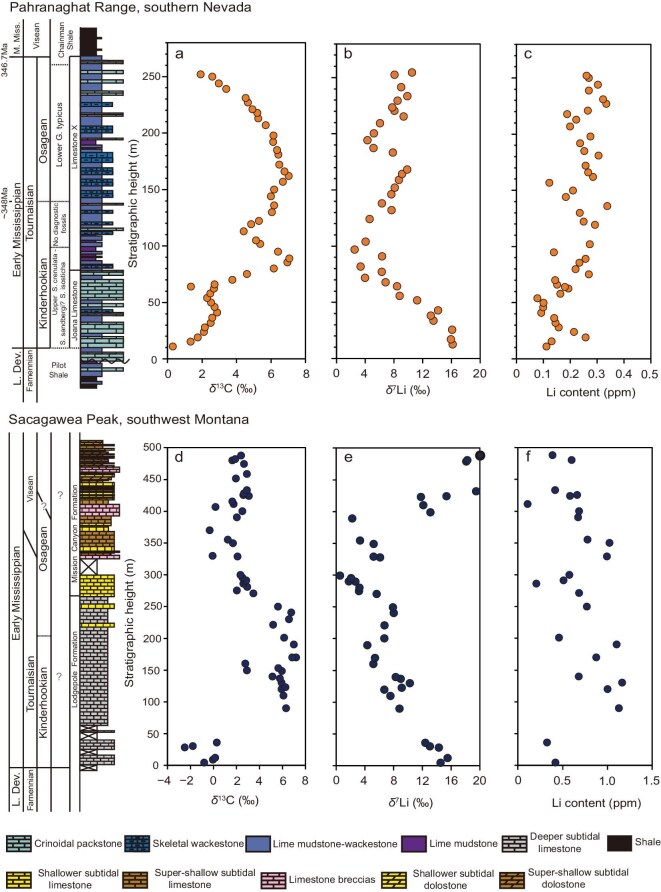
The results for δ^13^C, δ^7^Li, and Li content of carbonates in the PR and SP sections. For each study site (PR and SP), all isotope data were generated from the same suite of samples.

In the PR section, the stratigraphic trend of the δ^13^C_carb_ record reveals two prominent peaks within the TICE interval (Fig. 2). The δ^13^C_carb_ values initially start at a baseline of −0.31‰ at ∼11 m depth. This is followed by a significant rise to 7.06‰ at the first peak at 89 m and a second peak at 7.02‰ at 162 m. After the second peak, the δ^13^C_carb_ values gradually decline, returning toward baseline conditions. The δ^7^Li profile in the PR exhibits substantial and coherent stratigraphic variations, with values ranging from 2.2‰ to 16.1‰ and an average of 8.2‰ ± 7.2‰ (2SD). Two distinct negative excursions are observed: the first involves a sharp decrease from 16.1‰ at 11 meters to 2.2‰ at 95 m, followed by an increase to 9.6‰ between 95 and 166 m. This is subsequently followed by another decrease to the nadir of 4.0‰ at 192 m. After that, the δ^7^Li begins to recover but does not return to pre-excursion conditions.

In the SP section, both the δ^13^C_carb_ and δ^7^Li records show stratigraphic trends similar to those in the PR section. The δ^13^C_carb_ record, although exhibiting two peaks, has lower temporal resolution. The δ^7^Li ranges from 20.4‰ to 0.5‰, with an average of 8.8‰ ± 10.7‰ (2SD). The correlation between the two sections is based on strata boundaries and the trends in δ^13^C_carb_ data (Fig. S1). Overall, the parallel trends observed in δ^13^C_carb_ and δ^7^Li across both sections underscore the influence of global phenomena on these proxies during the TICE interval. Detailed geochemical data and age models are provided in the Supplementary information.

## DISCUSSION

### Evaluation of carbonate δ^7^Li as records of isotopic compositions of coeval seawater

The δ^7^Li signatures of inorganic carbonates are likely susceptible to contamination by detrital silicates in bulk samples, due to notably lower Li contents in carbonate minerals relative to silicate minerals. Thus, the molar ratios of Al/(Ca + Mg) of the studied samples are first examined to constrain this potential contribution. By using a sequential leaching approach with 1 M acetic acid, we find that all leached carbonate fractions are marked by low Al/(Ca + Mg) ratios (0.02–0.7 mmol/mol), significantly lower than the recommended threshold value of 0.8 mmol/mol in previous studies [35,37]. We further calculate the proportion of carbonate-hosted Li (Li_carb_) and their isotopes (δ^7^Li_carb_) by eliminating the potential addition of Li from the silicate minerals (see *Supplementary information* for calculation method). The results show high fractions of Li_carb_ in bulk samples and highly consistent trends of δ^7^Li_carb_ and δ^7^Li of the studied carbonates, providing additional evidence that large δ^7^Li_carb_ variations in the studied sections are not induced by detrital contamination during the sample leaching. Although appreciable correlations between δ⁷Li and Al/(Ca + Mg) are identified in both study sections (Figs S4 and S5), the SP section exhibits a positive correlation between δ⁷Li and Al/(Ca + Mg) (*R*^2^ = 0.29), inconsistent with the case that detrital silicates commonly contribute notably low δ⁷Li signals relative to carbonate mineral. As for PR section, a negative correlation can be observed between δ⁷Li and Al/(Ca + Mg) (*R*^2^ = 0.37). Nevertheless, most of the samples have very low Al/(Ca + Mg) ratios (<0.4 mmol/mol), which shows very limited contributions of detrital silicates. Here, we propose that negative correlations of δ⁷Li and Al/(Ca + Mg) may not reflect detrital contaminations during sample digestion, instead, can be interpreted by increased inputs of terrestrial detrital materials into the study section [38]. The δ^7^Li_carb_ signatures have also been considered relative to the carbonate mineralogy and diagenetic process, based on systematic studies of biogenic carbonates [39] and late Cenozoic inorganic carbonates [40–42]. Shallow-marine carbonates experiencing marine burial diagenesis generally show δ^7^Li_carb_ values approaching those of coeval seawater, following fluid-buffered conditions for marine diagenesis of the Li isotope system [40–42]. In contrast, primary carbonate sediments and meteorically altered carbonates exhibit large Li isotopic offsets relative to coeval seawater. The studied successions exhibit significant positive δ^13^C_carb_ excursions with relatively high δ^18^O_carb_ values, opposite to the typical characteristics for meteoric diagenetic carbonates that are marked by coupled negative excursions of δ^13^C_carb_ and δ^18^O_carb_ values. Although several samples in the SP and PR sections exhibit low δ^18^O_carb_ (< −10‰), most of samples in these two sections have relatively high δ^18^O_carb_ values between −6‰ and −2‰, and do not show appreciable correlations between δ⁷Li_carb_ and δ^18^O_carb_. Moreover, the samples with notably negative δ^18^O_carb_ in the SP section are marked by high δ⁷Li_carb_, rather than low δ⁷Li_carb_ values. Further, the studied carbonates show notably low molar ratios of Mg/(Ca + Mg) (0.01–0.05) and Sr/(Ca + Mg) (0.3–1.3) during the TICE interval, indicating that these carbonates are composed of low-Mg calcites more likely experiencing marine burial diagenesis [41,43]. Although very weak correlations of δ^7^Li_carb_ vs. Sr/(Ca + Mg) (*R*^2^ = 0.31, *P* < 0.05) and Li/(Ca + Mg) (*R*^2^ = 0.18, *P* < 0.05) can be observed in the studied carbonates, the main interval of negative δ^7^Li_carb_ excursions and positive δ^13^C_carb_ excursions shows relatively scattered and varying values of Sr/(Ca + Mg) and Li/(Ca + Mg) (Figs S4 and S5). Further, we examined the rare earth elements (REE) of both sections to exclude the additional effects of regional hydrothermal fluids and diagenetic alteration on δ^7^Li_carb_ values (Fig. S6). The studied two sections exhibit similar normalized REE patterns analogous to the typical seawater-like pattern without appreciable positive Eu anomaly (a commonly used proxy for hydrothermal fluids) [44]. Taken together, we suggest that coupled excursions of δ^7^Li_carb_ (∼12‰) and δ^13^C_carb_ (∼7‰) in the shallow-marine carbonates were not driven by changes in carbonate mineralogy and diagenetic alteration, and instead reflect large perturbations of global marine biogeochemical cycles and climate in the Early Mississippian as documented in the studied shallow-marine carbonates.

### Major Li isotope variations of global seawater and perturbations of continental silicate weathering during the TICE event

Understanding the temporal changes in continental silicate weathering is critical for evaluating the role of CO₂ drawdown in climatic transitions during the Early Mississippian. After rigorous screening for diagenetic alteration and clay contamination, as presented above, we adopt a conservative value of ∼−2‰ for Li isotopic fractionation between marine limestone and coeval seawater, based on the results of late Cenozoic carbonates from the Bahamas and South China Sea [40–42]. This approach aligns with empirical calibrations from modern Bahamian and South China Sea carbonates, where low-Mg calcite that underwent marine burial diagenesis exhibits relatively limited Li isotope offsets relative to coeval seawater under seawater buffered conditions [40–42]. Applying this offset, reconstructed seawater δ⁷Li values range from 6.2‰ to 20.1‰ in the PR section and from 4.5‰ to 24.4‰ in the SP section. The large negative δ⁷Li excursions in these two carbonate sections suggest dramatic decreases in global seawater δ⁷Li, in step with increased δ^13^C during the earliest Carboniferous. Under million-year time scales, global seawater δ⁷Li values can be regulated by changes in flux and isotopic compositions of river waters, mid-ocean ridge hydrothermal fluids, and authigenic clays [45,46]. The δ⁷Li values of mid-ocean ridge hydrothermal fluids are generally considered to remain constant with low values of ∼8‰ through time, due to the limited Li isotope fractionations during the effective basalt weathering at high temperatures [47]. Thus, only hydrothermal flux can have an appreciable influence on Li isotope mass balance of global ocean. For instance, increased input of hydrothermal fluids into the global ocean would decrease global seawater δ⁷Li values toward the hydrothermal value. However, changes in mid-ocean ridge hydrothermal flux are commonly related to seafloor spreading over longer tectonic time scales (>10 Myr), making it unlikely to drive a rapid δ⁷Li excursion of global seawater during the 4-Myr TICE [cf. 8,48]. Further, given an overall value of ∼8‰ for mid-ocean ridge hydrothermal fluid, seawater δ⁷Li values as low as 6.2‰–4.5‰ during the TICE event cannot solely be induced by enhanced hydrothermal inputs. Additionally, the estimated mid-ocean ridge hydrothermal fluxes gradually decreased during the early Carboniferous, based on secular changes in seawater chemistry [49,50]. All these observations argue against the case that rapid decreases in global seawater δ⁷Li are related to effects of the mid-ocean ridge hydrothermal fluids. The fractionation between marine authigenic clays and seawater also affect the global seawater δ⁷Li, as they generally remove isotopically light Li from seawater [34,37]. However, decreased sink fractionation, as would be required to explain reported negative δ⁷Li excursions, mostly correspond to enhanced marine inorganic Si inventory or increased temperatures that mute the overall isotopic fractionations between seawater and marine authigenic clays [34,45,51]. This is opposite to the environmental background during the TICE in the earliest Carboniferous (that is, global cooling and widespread biosilicification in oceans) [2, 52,53]. Here, we use a value of ∼−5‰ for Li isotopic offset between marine authigenic clays and seawater, representing the overall isotopic fractionations for global marine Li sinks during the late Paleozoic [37,54]. The value of −5‰ is already much lower than that in the late Cenozoic ocean [46], which reflects overall mute isotopic fractionations between Li sinks and the late Paleozoic seawater due to more quantitative removal of Li in marine sediments driven by high reverse weathering rates before the rise of diatoms. Thus, only decreasing Li isotope offsets between authigenic clays and seawater cannot account for negative δ⁷Li excursions of ∼12‰ during the TICE event. Taken together, we suggest that the rapid and large decreases in global seawater δ⁷Li during the TICE were not caused by enhanced mid-ocean ridge hydrothermal flux or decreased isotopic fractionation associated with marine authigenic clay formation. Instead, dramatic changes in global climate and accordingly continental silicate weathering are ultimately considered to account for the significant perturbations in the global oceanic Li isotope cycle.

The riverine Li flux and δ⁷Li values are highly determined by regional weathering intensity [20], reflecting the balance of primary silicate dissolution and formation of secondary clays. Based on modern riverine frameworks, medium weathering intensity favors extensive clay formation and high δ⁷Li values of river waters [20]. Conversely, low weathering intensity yields δ⁷Li of river waters closer to bedrock values and a high riverine Li flux [20]. The negative excursions of ∼12‰ in seawater δ⁷Li, inferred from the SR and PR records, require a substantial perturbation in the global marine Li isotope budgets by enhancing riverine Li flux with low δ⁷Li values, which reflects rapidly decreased weathering intensity during the TICE. Notable negative δ⁷Li excursions of global seawater have commonly occurred during the Phanerozoic, especially under warming climate, which reflect decreases in weathering intensity (for example, Cretaceous oceanic anoxic events, Paleocene–Eocene thermal maximum) [18,35,55]. Decreased weathering intensity in step with global warming generally suggests greater increases in the physical erosion rate than chemical weathering rate, likely due to an enhanced hydrological cycle and supply of fresh materials [18,35,56]. In contrast, oxygen isotopes demonstrate that the TICE was characterized by an important climate cooling [2,52]. Therefore, rapid decreases in weathering intensity at global scales more likely reflect variations of physical erosion rates triggered by a mechanism other than global climate change, as low atmospheric CO_2_ and surface temperature are unlikely to be able to enhance the global hydrological cycle and weathering kinetics. Hence, in this study, we suggest that enhanced erosion and chemical weathering rates—potentially driven by initial uplift of the Variscan range in the equatorial area [12,57,58] or the rise of seed land plants during the TICE event [8,59]—likely increased the supply of fresh silicate minerals to weathering zones, overwhelmed clay-forming reactions and reduced the effective fractionation between bedrock and dissolved Li, ultimately driving a large negative δ⁷Li excursion of global seawater.

### Modeling the Li–C isotope excursions

To further understand the factors and processes that controlled the coupled excursions in δ^13^C_carb_ and δ⁷Li_sw_ during the TICE, we use the global biogeochemical cycles model GEOCLIM [60,61] and COPSE [13,62], particularly focusing on the effects of enhanced uplift and land plant evolution on global continental silicate weathering. Previous studies have considered the effects of global continental weatherability and volcanic degassing on secular changes in atmospheric CO_2_ level and global climate from the late Devonian to the carboniferous, using COPSE and SCION models [10,13]. While more modeling tests on different processes (for example, degassing rate, physical erosion, and land plant evolution) are required to independently constrain the evolution of continental silicate weathering and its effect on atmospheric CO_2_ levels, and ultimately reproduce the coupled variations in isotopic records in marine sediments during the TICE event.

**Figure 3.**
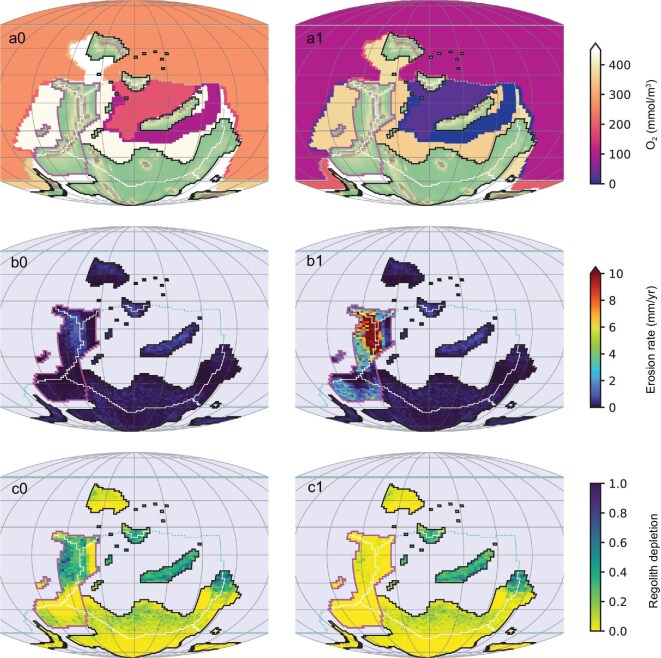
Maps of GEOCLIM simulation (two time slices). ‘0’ maps (left column) are the initial condition, ‘1’ maps (right column) are at the perturbation peak (3 Myr of run time, cf. Fig. 4a). (a0–1) Topography and oceanic O_2_ concentration at bottom of water column, (b0–1) physical erosion rate, showing enhanced physical erosion around the Variscan orogeny, and (c0–1) regolith depletion quantified by the proportion of unweathered mineral at top of regolith. The uplifted area is contoured in magenta on each map. The boundaries of oceanic boxes, and their corresponding continental drainage basins are drawn on each map (solid and dotted lines). GEOCLIM computes one mean O_2_ concentration for each of the 21 boxes. The maps panels (a0–1) show the mean concentrations of GEOCLIM bottom boxes drawn on the full geographic extent of these boxes at the oceanic resolution of the FOAM ocean-atmosphere general circulation model [87] simulations, which were used as GEOCLIM forcing.

The GEOCLIM model simulates the C, O, and S cycles (including marine redox state) by spatially resolving (Fig. 3) the oceanic and continental processes that control the budget of the above-mentioned elements. Compared to most long-term carbon cycle models (for example, COPSE and LOSCAR), GEOCLIM has the unique capacity to simulate the influence of physical erosion on continental weathering, based on spatially resolved climatic and topographic information provided by general circulation models and paleogeographical reconstructions [61,63]. GEOCLIM also simulates the C isotopic cycle [60,61]. For this study, we implemented the Li isotopic cycle in GEOCLIM, including its relation to weathering intensity [18,20,30], using the model of Caves Rugenstein *et al.* [64] (see Methods).

**Figure 4.**
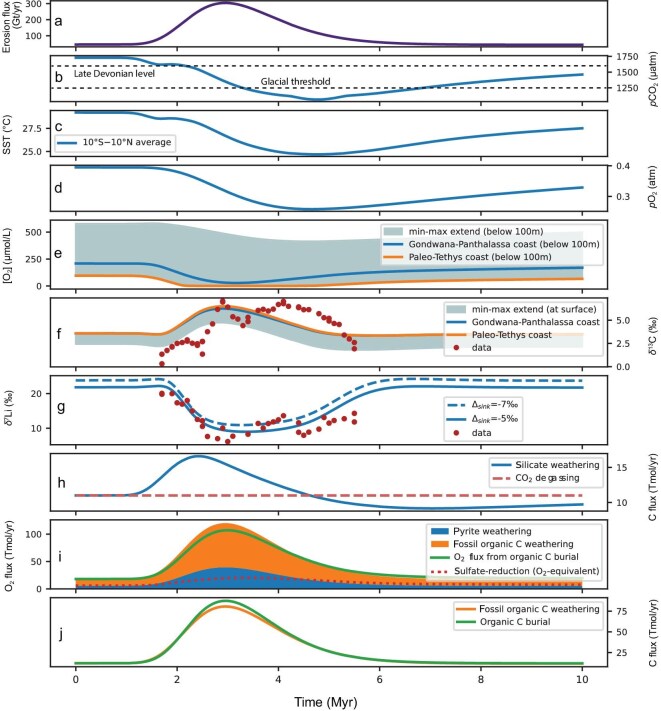
Full time-series of GEOCLIM simulation. (a) Global erosion flux; (b) atmospheric *p*CO_2_; (c) sea surface temperature near the equator (deduced from the general circulation model simulations used as GEOCLIM forcing); (d) atmospheric *p*O_2_; (e) oceanic O_2_ concentration in non-surface (that is, below 100 m) GEOCLIM boxes, minimum–maximum range and two selected boxes; (f) δ^13^C of oceanic dissolved inorganic C in GEOCLIM surface boxes, minimum–maximum range and two selected boxes, surface dissolved δ^13^C is representative of what is precipitated in carbonates, and preserved in sediments; (g) mean seawater δ^7^Li from GEOCLIM and recomputed with a smaller authigenic clay fractionation (*Δ*_sink_); (h) inorganic C source (magmatic degassing) and sink (silicate weathering); (i) O_2_ source (organic C burial) and sinks (oxidative weathering, stacked), sulfate-reduction is not an O_2_ flux, and is only shown to be compared to pyrite weathering, using the same conversion factor; and (j) organic C source (fossil organic C weathering) and sink (organic C burial). The two selected GEOCLIM boxes on panels (e) and (f) are the ‘Gondwana-Panthalassa coast’ box where data locations are situated (see maps of Figs 1 and 3) and the ‘paleo-Tethys’ coast box where the anoxia is the most pronounced. The data of the PR section are reported on panels (f) and (g), assuming a seawater–limestone δ^7^Li offset of 2‰.

We test here the scenario whereby the environmental changes during the TICE are caused by increases in continental physical erosion associated with the initial development of Variscan orogeny in the equatorial area [57,58]. Such scenario was hinted by the need of a shift of weathering intensity to explain the δ⁷Li_sw_ negative excursion, and the fact that a decreased weathering intensity, due to increased physical erosion, is likely to generate climate cooling, while an increased weathering intensity is not [65]. The strength of our experiment is to simply impose an erosion perturbation based on the negative δ⁷Li_sw_ excursions, but not to independently prescribe any weathering flux or δ⁷Li signature, which are mechanistically predicted by the model, as well as the ensuing evolution of the intertwined geochemical cycles, oceanic oxygen, and carbon isotopes. We do not use the updated paleogeographic reconstruction in recent studies [13,66] that focused on shifts in global continental weatherability due to increased land availability in the high weathering zones. Instead, the originally typical paleogeopgraphic landscape [67] was employed to specifically explore the effects of uplift and physical erosion on continental chemical weathering. Nevertheless, we conducted a sensitivity test to evaluate the effect of the area extent of inundated shelves, that is the most significant difference between the two reconstructions (see Supplementary information). Corresponding to initial uplift of Variscan range roughly coincident with the TICE event [57,58], the erosion perturbation was localized in a broad area that encompasses the Variscan range (magenta contour in Fig. 4). Its temporal evolution was designed as a lognormal pulse (see Fig. 4a, showing the global Earth-integrated erosion flux), whose duration matches the TICE interval (∼3 Myr) and whose amplitude is set to obtain the observed ∼12‰ negative δ⁷Li_sw_ excursion (Fig. 4g). The details of the spatio-temporal implementation of the erosion perturbations in GEOCLIM are provided in the Supplementary information. As consequences of the erosion pulse, the model shows a significant reduction of regolith depletion in the uplifted zone (Fig. 3 c0 to c1), indicating a reduced weathering intensity on global scales. A erosion-driven decrease in weathering intensity also accompanies with increased chemical weathering rate [25,68], and yet more efficient weathering [69], leading to enhanced CO_2_ drawdown and global cooling (Fig. 4b, c). Erosion also enhances oxidative weathering (fossil organic C and sulfides), which are CO_2_ sources, and partially offset the global cooling, maintaining the global silicate weathering flux up to 50% higher than its initial value (Fig. 4i), which would not have been possible without an additional C source [70]. This substantial silicate weathering peak is associated with a nutrient pulse, fostering oceanic primary productivity, deoxygenation, and organic C burial (Fig. 4f and j). Sulfate reduction, on the other hand, exhibits a delayed rise (Fig. 4i), as it is (partially) limited by SO_4_^2−^, whose residence time is much longer than the TICE interval (tens of Myr). This peculiarity has a crucial consequence: organic C burial does not totally offset the oxidative weathering fluxes (Fig. 4i), enabling anoxia by lowering atmospheric O_2_ (Fig. 4d) and sustaining the nutrient pulse. Yet, because of the lack of organic C remineralization by sulfate reduction, organic C burial does exceed fossil organic C weathering (Fig. 4j), leading to a positive δ^13^C_carb_ excursion (Fig. 4f).

In summary, the synergistic effects of shifting the silicate weathering intensity, enhancing the oxidative weathering fluxes, and the interweaving of C, O, and S cycles explain how a δ⁷Li_sw_ excursion is also consistent with observed δ^13^C_carb_ excursion, global cooling and marine anoxia—based on conodont and brachiopod O isotope records [8,2,52] and carbonate U isotope records [11]. Our results provide a mechanistic link between Devonian greenhouse conditions and the onset of Carboniferous icehouse conditions. We did not make any effort to fully fit the δ^13^C_carb_ excursion, and we note that the one predicted by GEOCLIM is weaker than the observations δ^13^C_carb_ records (Fig. 4f). This suggests that there may be missing processes in GEOCLIM that would amplify the excursion. One possible process could be a stronger P-anoxia positive feedback, that would amplify the organic C burial. A recent study using a cGENIE model highlighted the significance of this feedback [71], which is indeed stronger in their model than in GEOCLIM model [61]. Another possibility is that the uplift perturbation would increase more sulfide weathering that it increases fossil organic C weathering, leading to further deoxygenation (and organic C burial) without light ^13^C input. This is plausible since fossil organic C weathering was suggested to have some kinetic limitation at very high erosion rates [72]. Finally, our sensitivity test (see Supplementary information and Fig. S13) suggests that a large fraction of inundated shelves is likely needed to explain the amplitude of the δ^13^C_carb_ excursion—in line with the paleogeographical reconstruction of the PALEOMAP project used in our main simulation.

**Figure 5.**
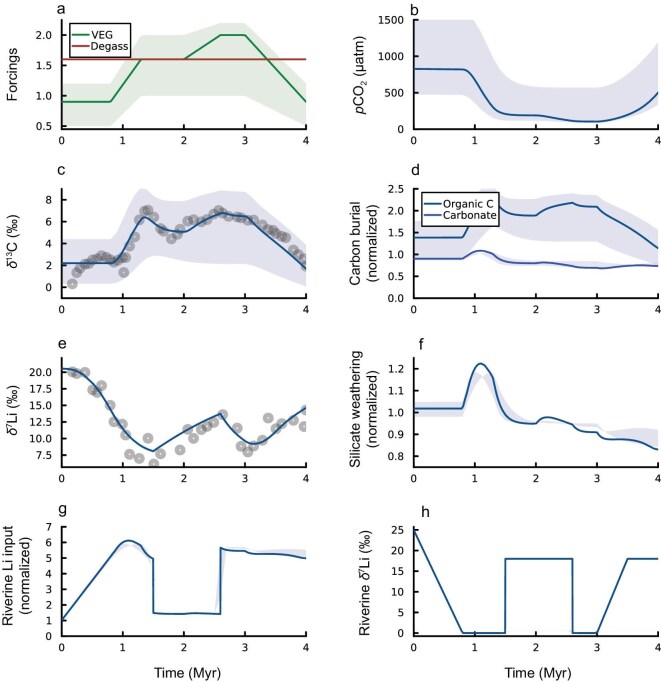
Biogeochemical modelling results. (a) COPSE forcings Vegetation (VEG, bar shows the sensitivity tests). VEG = [−1e30, −350.2e6, −349.7e6, −349.0e6, −348.4e6, −348e6, −347e6, 1e30], [Vbaseline, Vbaseline, Vpeak1, Vpeak1, Vpeak2, Vpeak1, 1.0]. Here, the first vector is the time in years, and the second shows forcing values at these times. Vbaseline = (0.5, 0.9, 1.2), Vpeak1 = (1.0, 1.6, 2.0), Vpeak2 = (1.0, 2.0, 2.2). Degassing (D). (b) Atmospheric *p*CO_2_ (μatm). (c) Model-data comparison of δ^13^C_carb_. (d) Normalized carbonate and organic carbon (both terrestrial and marine-derived material) burial fluxes. (e) Model-data comparison of δ^7^Li. (f) Normalized silicate weathering flux. (g) Normalized riverine lithium input flux. (h) Isotopic values of the riverine lithium input. The bars in (b–d, f) show the responses of the sensitivity tests on the VEG.

Alternatively, previous studies indicate that the Tournaisian seed-plant floras underwent major diversification [8], which likely triggered increases in chemical weathering [73,74]. Changes in δ^13^C_carb_ and δ⁷Li_sw_ may also be reproduced using the COPSE model via the implementation of an increase in COPSE’s vegetation forcing from a pre-plant baseline (0.9 relative to modern level) to higher than modern levels (1.6–2.0) (Fig. 5). This approach highlights the feedback mechanisms of the carbon cycle: over ∼1 million years, the expansion of vegetation leads to a significant increase in organic carbon burial (∼1.5 times), driving the δ^13^C_carb_ excursion and reducing *p*CO₂ from about 1000 ppmv to roughly 200 ± 200 ppmv. Concurrently, silicate weathering rate increases modestly (∼1.3 times, from 0.15 to 0.2 of modern levels) in COPSE, contributing to the riverine Li flux and the negative δ⁷Li_sw_ excursion. It is acknowledged that COPSE model has not fully considered the changes in physical erosion, and weathering intensity that is directly linked to riverine δ⁷Li values, while increased silicate weathering rates via enhanced colonization of land plants, in part, contribute to increased riverine Li flux and decreased δ⁷Li [20]. Taken together, in additional to previous evaluation of effects of paleogeographic locations on global chemical weathering, enhanced physical erosion and subsequently increased chemical weathering, associated with widespread uplift or enhanced colonization of land plants may have provided the plausible hypotheses for global cooling from the earliest Carboniferous, and reliably reproduced the coupled changes in global δ^13^C_carb_ and δ⁷Li_sw_ observed during the TICE event.

### Implications of enhanced weathering during the Early Mississippian, Carboniferous

Our δ⁷Li data and model outputs have significant implications for the evolution of the surface Earth system during the Early Mississippian, Carboniferous. The ∼12‰ decrease in global seawater δ⁷Li requires the riverine Li flux to increase roughly two to four times with low riverine δ⁷Li values during the TICE event. These changes in weathering intensity, accompanied by coupled increases in physical erosion and chemical weathering rates would have delivered nutrients to the oceans, stimulated marine productivity, and led to the expansion of marine anoxia. Evidence from nitrogen, zinc, and barium isotopes during the TICE intervals supports increased primary productivity in Early Mississippian oceans [14–16]. Enhanced productivity would have resulted in the expansion of anoxia despite cooling climatic conditions, as confirmed by uranium isotope data from the PR section [11], the major positive carbon isotope excursion and our modeling results (Figs 3 and 4). Recently published papers have indicated that volcanic carbon degassing flux was close to present-day levels in the early Carboniferous [10], and development of large igneous provinces might have limited cooling effect on long-term climate during the late Paleozoic [75]. Thus, we suggest that the combined effects of enhanced silicate weathering due to enhanced physical erosion and marine productivity likely played critical roles in rapidly sequestrating atmospheric CO_2_ and ultimately climatic cooling, as indicated by rising δ^18^O_apatite_ and δ^18^O_conodont_ values across the TICE interval [8,2,76,77]. Although climate cooling may have occurred since the late Devonian, independent proxy data suggests atmospheric *p*CO₂ levels are still above glacial threshold (for example, <1120 ppmv) during the Devonian–Carboniferous transition [12,78,79]. Our model results, which show a further decrease in atmospheric *p*CO₂ during the TICE event from an initial level of around 1400 ppmv to below 400 ppmv—comparable to modern Earth conditions—thus allow ice accumulation in the polar regions, and facilitate the onset of the LPIA [78–80].

## METHODS

### Sampling and analysis

We studied two coeval carbonate sections from the North America: the PR section in southern Nevada and the SP section in southwest Montana. We sampled the Lower Mississippian Joana Limestone and Limestone X from the PR section. The stratigraphy at this location has been described in detail by Saltzman *et al.* [81] and Maharajan *et al.* [7]. Briefly, the 86-m-thick Joana Limestone is dominated by crinoid-rich grainstone and packstone (representing upper shoreface environments), and the overlying 186-m-thick Limestone X consists mainly of lime mudstone and wackestone, with subordinate thin packstone and grainstone storm interbeds (representing offshore to lower shoreface environments). All two sections have been extensively studied for carbon isotope stratigraphy and conodont biostratigraphy by Saltzman *et al.* [81,3], Maharajan *et al.* [7], and Cheng *et al.* [11] providing a high-resolution temporal framework for the correlations between the two localities. Forty seven samples from the PR section and 30 samples from the SP section were analyzed for δ^7^Li and major and trace elements at the Centre for Research and Education on Biological Evolution and Environment Nanjing University, following the typical procedure of carbonate Li isotope analysis in Wei *et al.* [42]. See detailed information of analytical methods and results in Supplementary information.

### Description of the model coupling between Li–C isotopes and GEOCLIM

The numerical model GEOCLIM is a coupled Earth system model for long-term climate and biogeochemical cycle, which works as a ‘bridge’ between global biogeochemical box models and Earth system models [60,61,82]. In this study, a version 7.0 of GEOCLIM [61] is used to evaluate the changes in global erosion and weathering evolution and C–Li isotope cycles during the TICE event. See detailed model descriptions and runs in the Supplementary information.

### Description of the model coupling between Li–C isotopes and COPSE

The box model is an extended version of the carbon, oxygen, phosphorus, sulfur, and evolution (COPSE) Earth system model with extended lithium cycle [17,62,83]. The model incorporates Li reservoirs and the isotopic mass balance equations for C and Li isotopes as tracers of the predicted outcomes to evaluate the influence of the weathering intensity on the coupled C–Li cycles. In this study, the offset between carbonate and seawater is set to 2 ‰ (δ⁷Li_seawater_ − δ⁷Li_carb_ = 2‰), the fractionation between authigenic clays to seawater is set to −5 ‰ (δ⁷Li_seawater_ − δ⁷Li_clay_ = 5‰). We should note that the COPSE model has limitation in describing the fractionation of riverine Li isotopes due to the changes in weathering intensity. Therefore, we modified the δ⁷Li_riverine_ and the flux of riverine Li input directly to reproduce the negative δ⁷Li_seawater_ excursions.

Early Mississippian steady state: The ∼351 Ma steady state has ocean lithium ≅1 of present ocean level (26e-3 mol/kg), *p*CO_2_ ≅ 1000 (μatm), the *p*O_2_ ≅ 0.35 (% atm). This steady state has much higher *p*CO_2_ level than the standard COPSE reloaded version [62]. To achieve this, we modified the physical forcings ‘Uplift’ and ‘Degass’ to increase the source of the atmosphere-ocean carbon reservoir, regulating the system at *p*CO_2_ ≅1000 (μatm). The other physical forcings inherited from the GEOCARB and COPSE models [62,83,84] are fixed at values for the Mississippian (351 Ma). To obtain the ∼2 ‰ background δ^13^C_carb_ signal, we assume δ^13^C values of 0.0 ‰ and −24 ‰ for sedimentary rock reservoirs of organic (reduced) carbon and carbonate (oxidized) carbon, respectively. See detailed model description, modification in the forcings and key parameters (Table S3) and four groups of extended model runs (Figs S6–S9) in the Supplementary information.

## Supplementary Material

nwag168_Supplemental_File

## Data Availability

All data needed to evaluate the conclusions in the paper are present in the paper and/or the *Supplementary Information*. GEOCLIM code, input data, and configuration files associated with the article: https://doi.org/10.5281/zenodo.16406861. Climate model (FOAM) outputs and GEOCLIM outputs associated with the article: https://doi.org/10.5281/zenodo.16410946. The COPSE model configurations were developed using the PALEOtoolkit (https://github.com/PALEOtoolkit), with adaptors to interface with the advanced equation solvers [85] in Julia programming language [86].

## References

[1] Montañez IP, Poulsen CJ. The late Paleozoic Ice age: an evolving paradigm. Annu Rev Earth Planet Sci 2013; 41: 629–56. 10.1146/annurev.earth.031208.100118

[2] Buggisch W, Joachimski MM, Sevastopulo G et al. Mississippian δ^13^C_carb_ and conodont apatite δ^18^O records—their relation to the late Palaeozoic glaciation. Palaeogeogr Palaeoclimatol Palaeoecol 2008; 268: 273–92. 10.1016/j.palaeo.2008.03.043

[3] Saltzman MR . Carbon and oxygen isotope stratigraphy of the Lower Mississippian (Kinderhookian–lower Osagean), western United States: implications for seawater chemistry and glaciation. Geol Soc Am Bull 2002; 114: 96–108. 10.1130/0016-7606(2002)114<0096:CAOISO>2.0.CO;2

[4] Saltzman MR, Groessens E, Zhuravlev AV. Carbon cycle models based on extreme changes in δ^13^C: an example from the lower Mississippian. Palaeogeogr Palaeoclimatol Palaeoecol 2004; 213: 359–77. 10.1016/j.palaeo.2004.07.019

[5] Braun MG, Anderson NT, Bergmann KD et al. Early Mississippian global δ^13^C excursion is not a diagenetic artifact. Geology 2024; 52: 641–5. 10.1130/G52109.1

[6] Yao L, Qie W, Luo G et al. The TICE event: perturbation of carbon–nitrogen cycles during the mid-Tournaisian (Early Carboniferous) greenhouse–icehouse transition. Chem Geol 2015; 401: 1–14. 10.1016/j.chemgeo.2015.02.021

[7] Maharjan D, Jiang G, Peng Y et al. Sulfur isotope change across the early Mississippian K–O (Kinderhookian–Osagean) δ^13^C excursion. Earth Planet Sci Lett 2018; 494: 202–15. 10.1016/j.epsl.2018.04.043

[8] Chen B, Chen J, Qie W et al. Was climatic cooling during the earliest Carboniferous driven by expansion of seed plants? Earth Planet Sci Lett 2021; 565: 116953. 10.1016/j.epsl.2021.116953

[9] Mii H-s, Grossman EL, Yancey TE. Carboniferous isotope stratigraphies of North America: implications for Carboniferous paleoceanography and Mississippian glaciation. Geol Soc Am Bull 1999; 111: 960–73. 10.1130/0016-7606(1999)111<0960:CISONA>2.3.CO;2

[10] Merdith AS, Gernon TM, Maffre P et al. Phanerozoic icehouse climates as the result of multiple solid-Earth cooling mechanisms. Sci Adv 2025; 11: eadm9798. 10.1126/sciadv.adm9798 39951538 PMC11827867

[11] Cheng K, Elrick M, Romaniello SJ. Early Mississippian ocean anoxia triggered organic carbon burial and late Paleozoic cooling: evidence from uranium isotopes recorded in marine limestone. Geology 2020; 48: 363–7. 10.1130/G46950.1

[12] Goddéris Y, Donnadieu Y, Carretier S et al. Onset and ending of the late Palaeozoic ice age triggered by tectonically paced rock weathering. Nat Geosci 2017; 10: 382–6. 10.1038/ngeo2931

[13] Marcilly CM, Torsvik TH, Jones MT. Late Paleozoic climate transition from a long-term carbon cycle modeling perspective. Glob Planet Change 2025; 253: 104984. 10.1016/j.gloplacha.2025.104984

[14] Zhong Y, Chen J, Liu S-A et al. Zinc isotope perspective on global carbon cycling during the onset of the late Paleozoic icehouse. Geology 2024; 53: 99–104. 10.1130/G52447.1

[15] Zhang F, Pohl A, Elrick M et al. Enhanced marine biological pump as a trigger for the onset of the late Paleozoic ice age. Sci Adv 2025; 11: eadv2756. 10.1126/sciadv.adv2756 40601752 PMC12219544

[16] Liu J, Algeo TJ, Qie W et al. Intensified oceanic circulation during early Carboniferous cooling events: evidence from carbon and nitrogen isotopes. Palaeogeogr Palaeoclimatol Palaeoecol 2019; 531: 108962. 10.1016/j.palaeo.2018.10.021

[17] Pogge von Strandmann PAE, Desrochers A, Murphy MJ et al. Global climate stabilisation by chemical weathering during the Hirnantian glaciation. Geochem Perspect Lett 2017; 3: 230–7. 10.7185/geochemlet.1726

[18] Pogge von Strandmann PAE, Jones MT, West AJ et al. Lithium isotope evidence for enhanced weathering and erosion during the Paleocene–Eocene Thermal Maximum. Sci Adv 2021; 7: eabh4224. 10.1126/sciadv.abh4224 34652934 PMC8519576

[19] Pogge von Strandmann PAE, Burton KW, Opfergelt S et al. The effect of hydrothermal spring weathering processes and primary productivity on lithium isotopes: lake Myvatn, Iceland. Chem Geol 2016; 445: 4–13. 10.1016/j.chemgeo.2016.02.026

[20] Dellinger M, Gaillardet J, Bouchez J et al. Riverine Li isotope fractionation in the Amazon River basin controlled by the weathering regimes. Geochim Cosmochim Acta 2015; 164: 71–93. 10.1016/j.gca.2015.04.042

[21] Sauzéat L, Rudnick RL, Chauvel C et al. New perspectives on the Li isotopic composition of the upper continental crust and its weathering signature. Earth Planet Sci Lett 2015; 428: 181–92. 10.1016/j.epsl.2015.07.032

[22] Rudnick RL, Tomascak PB, Njo HB et al. Extreme lithium isotopic fractionation during continental weathering revealed in saprolites from South Carolina. Chem Geol 2004; 212: 45–57. 10.1016/j.chemgeo.2004.08.008

[23] Wimpenny J, James RH, Burton KW et al. Glacial effects on weathering processes: new insights from the elemental and lithium isotopic composition of West Greenland rivers. Earth Planet Sci Lett 2010; 290: 427–37. 10.1016/j.epsl.2009.12.042

[24] Wimpenny J, Colla CA, Yu P et al. Lithium isotope fractionation during uptake by gibbsite. Geochim Cosmochim Acta 2015; 168: 133–50. 10.1016/j.gca.2015.07.011

[25] West A, Galy A, Bickle M. Tectonic and climatic controls on silicate weathering. Earth Planet Sci Lett 2005; 235: 211–28. 10.1016/j.epsl.2005.03.020

[26] Gabet EJ, Mudd SM. A theoretical model coupling chemical weathering rates with denudation rates. Geology 2009; 37: 151–4. 10.1130/G25270A.1

[27] Marriott CS, Henderson GM, Crompton R et al. Effect of mineralogy, salinity, and temperature on Li/Ca and Li isotope composition of calcium carbonate. Chem Geol 2004; 212: 5–15. 10.1016/j.chemgeo.2004.08.002

[28] Pogge von Strandmann PAE, Frings PJ, Murphy MJ. Lithium isotope behaviour during weathering in the Ganges Alluvial Plain. Geochim Cosmochim Acta 2017; 198: 17–31. 10.1016/j.gca.2016.11.017

[29] Pogge von Strandmann PAE, Dellinger M, West AJ. Lithium Isotopes: a Tracer of Past and Present Silicate Weathering. Cambridge: Cambridge University Press, 2021. 10.1017/9781108990752

[30] Pogge von Strandmann PAE, Kasemann SA, Wimpenny JB. Lithium and Lithium isotopes in Earth’s surface cycles. Elements 2020; 16: 253–8. 10.2138/gselements.16.4.253

[31] Wei G-Y, Wei W, Wang D et al. Enhanced chemical weathering triggered an expansion of euxinic seawater in the aftermath of the Sturtian glaciation. Earth Planet Sci Lett 2020; 539: 116244. 10.1016/j.epsl.2020.116244

[32] Wei G-Y, Zhao M, Sperling EA et al. Lithium isotopic constraints on the evolution of continental clay mineral factory and marine oxygenation in the earliest Paleozoic era. Sci Adv 2024; 10: eadk2152. 10.1126/sciadv.adk2152 38552018 PMC10980266

[33] Sun H, Xiao Y, Gao Y et al. Rapid enhancement of chemical weathering recorded by extremely light seawater lithium isotopes at the Permian-Triassic boundary. Proc Natl Acad Sci USA 2018; 115: 3782–7. 10.1073/pnas.1711862115 29581278 PMC5899431

[34] Cao C, Bataille CP, Song H et al. Persistent late Permian to Early Triassic warmth linked to enhanced reverse weathering. Nat Geosci 2022; 15: 832–8. 10.1038/s41561-022-01009-x

[35] Pogge von Strandmann PAE, Jenkyns HC, Woodfine RG. Lithium isotope evidence for enhanced weathering during Oceanic Anoxic Event 2. Nat Geosci 2013; 6: 668–72. 10.1038/ngeo1875

[36] Wei G-Y, Pohl A, Jiang S et al. Changes in continental weathering regimes inhibited global marine deoxygenation during the Paleocene–Eocene Thermal Maximum. Nat Commun 2025; 16: 9163. 10.1038/s41467-025-64217-0 41093843 PMC12528694

[37] Kalderon-Asael B, Katchinoff JAR, Planavsky NJ et al. A lithium-isotope perspective on the evolution of carbon and silicon cycles. Nature 2021; 595: 394–8. 10.1038/s41586-021-03612-1 34262211

[38] Zhao M, Zheng Y. Marine carbonate records of terrigenous input into paleotethyan seawater: geochemical constraints from Carboniferous limestones. Geochim Cosmochim Acta 2014; 141: 508–31. 10.1016/j.gca.2014.07.001

[39] Dellinger M, West AJ, Paris G et al. The Li isotope composition of marine biogenic carbonates: patterns and mechanisms. Geochim Cosmochim Acta 2018; 236: 315–35. 10.1016/j.gca.2018.03.014

[40] Dellinger M, Hardisty DS, Planavsky NJ et al. The effects of diagenesis on lithium isotope ratios of shallow marine carbonates. Am J Sci 2020; 320: 150–84. 10.2475/02.2020.03

[41] Murphy JG, Ahm A-SC, Swart PK et al. Reconstructing the lithium isotopic composition (δ^7^Li) of seawater from shallow marine carbonate sediments. Geochim Cosmochim Acta 2022; 337: 140–54. 10.1016/j.gca.2022.09.019

[42] Wei G-Y, Zhang F, Yin Y-S et al. A 13 million-year record of Li isotope compositions in island carbonates: constraints on bulk inorganic carbonate as a global seawater Li isotope archive. Geochim Cosmochim Acta 2023; 344: 59–72. 10.1016/j.gca.2023.01.013

[43] Higgins JA, Blättler CL, Lundstrom EA et al. Mineralogy, early marine diagenesis, and the chemistry of shallow-water carbonate sediments. Geochim Cosmochim Acta 2018; 220: 512–34. 10.1016/j.gca.2017.09.046

[44] Tostevin R, Shields GA, Tarbuck GM et al. Effective use of cerium anomalies as a redox proxy in carbonate-dominated marine settings. Chem Geol 2016; 438: 146–62. 10.1016/j.chemgeo.2016.06.027

[45] Li G, West AJ. Evolution of Cenozoic seawater lithium isotopes: coupling of global denudation regime and shifting seawater sinks. Earth Planet Sci Lett 2014; 401: 284–93. 10.1016/j.epsl.2014.06.011

[46] Misra S, Froelich PN. Lithium isotope history of Cenozoic seawater: changes in silicate weathering and reverse weathering. Science 2012; 335: 818–23. 10.1126/science.1214697 22282473

[47] Chan L-H, Edmond JM, Thompson G. A lithium isotope study of hot springs and metabasalts from Mid-Ocean Ridge Hydrothermal Systems. J Geophys Res: Solid Earth 1993; 98: 9653–9. 10.1029/92JB00840

[48] Bruckschen P, Oesmann S, Veizer J. Isotope stratigraphy of the European Carboniferous: proxy signals for ocean chemistry, climate and tectonics. Chem Geol 1999; 161: 127–63. 10.1016/S0009-2541(99)00084-4

[49] Hardie LA . Secular variantion in seawater chemistry: an explanation for the coupled secular variation in the mineralogies of marine limestone and potash evaporites over the past 600 m.y. Geology 1996; 24: 279–83. 10.1130/0091-7613(1996)024<0279:SVISCA>2.3.CO;2

[50] Antonelli MA, Pester NJ, Brown ST et al. Effect of paleoseawater composition on hydrothermal exchange in midocean ridges. Proc Natl Acad Sci USA 2017; 114: 12413–8. 10.1073/pnas.1709145114 29109295 PMC5703293

[51] Coogan LA, Gillis KM, Pope M et al. The role of low-temperature (off-axis) alteration of the oceanic crust in the global Li-cycle: insights from the Troodos ophiolite. Geochim Cosmochim Acta 2017; 203: 201–15. 10.1016/j.gca.2017.01.002

[52] Grossman EL, Yancey TE, Jones TE et al. Glaciation, aridification, and carbon sequestration in the Permo-Carboniferous: the isotopic record from low latitudes. Palaeogeogr Palaeoclimatol Palaeoecol 2008; 268: 222–33. 10.1016/j.palaeo.2008.03.053

[53] Conley DJ, Frings PJ, Fontorbe G et al. Biosilicification drives a decline of dissolved Si in the oceans through geologic time. Front Mar Sci 2017; 4. 10.3389/fmars.2017.00397

[54] Liu X, Krause AJ, Wilson DJ et al. Lithium isotope evidence shows Devonian afforestation may have significantly altered the global silicate weathering regime. Geochim Cosmochim Acta 2025; 396: 107–21. 10.1016/j.gca.2025.02.036

[55] Lechler M, Pogge von Strandmann PAE, Jenkyns HC et al. Lithium-isotope evidence for enhanced silicate weathering during OAE 1a (Early Aptian Selli event). Earth Planet Sci Lett 2015; 432: 210–22. 10.1016/j.epsl.2015.09.052

[56] Krause AJ, Sluijs A, van der Ploeg R et al. Enhanced clay formation key in sustaining the Middle Eocene climatic Optimum. Nat Geosci 2023; 16: 730–8. 10.1038/s41561-023-01234-y 37564379 PMC10409649

[57] Cawood PA, Buchan C. Linking accretionary orogenesis with supercontinent assembly. Earth-Sci Rev 2007; 82: 217–56. 10.1016/j.earscirev.2007.03.003

[58] Rodríguez J, Cosca MA, Gil Ibarguchi JI et al. Strain partitioning and preservation of ^40^Ar/^39^Ar ages during variscan exhumation of a subducted crust (Malpica–Tui complex, NW Spain). Lithos 2003; 70: 111–39. 10.1016/S0024-4937(03)00095-1

[59] Algeo TJ, Berner RA, Maynard JB et al. Late Devonian oceanic anoxic events and biotic crises: “rooted” in the evolution of vascular land plants? GSA Today 1995; 5: 64–6.

[60] Goddéris Y, Joachimski MM. Global change in the Late Devonian: modelling the Frasnian–Famennian short-term carbon isotope excursions. Palaeogeogr Palaeoclimatol Palaeoecol 2004; 202: 309–29. 10.1016/S0031-0182(03)00641-2

[61] Maffre P, Goddéris Y, Le Hir G et al. GEOCLIM7, an Earth system model for multi-million-year evolution of the geochemical cycles and climate. Geosci Model Dev 2025; 18: 6367–413. 10.5194/gmd-18-6367-2025

[62] Lenton TM, Daines SJ, Mills BJW. COPSE reloaded: an improved model of biogeochemical cycling over Phanerozoic time. Earth-Sci Rev 2018; 178: 1–28. 10.1016/j.earscirev.2017.12.004

[63] Maffre P, Godderis Y, Pohl A et al. The complex response of continental silicate rock weathering to the colonization of the continents by vascular plants in the Devonian. Am J Sci 2022; 322: 461–92. 10.2475/03.2022.02

[64] Caves Rugenstein JK, Ibarra DE, von Blanckenburg F. Neogene cooling driven by land surface reactivity rather than increased weathering fluxes. Nature 2019; 571: 99–102. 10.1038/s41586-019-1332-y 31270485

[65] Froelich F, Misra S. Was the late Paleocene-early Eocene hot because Earth was flat? An ocean lithium isotope view of mountain building, continental weathering, carbon dioxide, and Earth’s Cenozoic climate. Oceanography 2014; 27: 36–49. 10.5670/oceanog.2014.06

[66] Marcilly CM, Torsvik TH, Domeier M et al. New paleogeographic and degassing parameters for long-term carbon cycle models. Gondwana Res 2021; 97: 176–203. 10.1016/j.gr.2021.05.016

[67] Scotese CR, Wright N. PALEOMAP paleodigital elevation models (PaleoDEMS) for the Phanerozoic. https://zenodo.org/records/5460860 (31 March 2026, date last accessed).

[68] West AJ . Thickness of the chemical weathering zone and implications for erosional and climatic drivers of weathering and for carbon-cycle feedbacks. Geology 2012; 40: 811–4. 10.1130/G33041.1

[69] Maffre P, Ladant J-B, Moquet J-S et al. Mountain ranges, climate and weathering. Do orogens strengthen or weaken the silicate weathering carbon sink? Earth Planet Sci Lett 2018; 493: 174–85. 10.1016/j.epsl.2018.04.034

[70] Berner RA, Caldeira K. The need for mass balance and feedback in the geochemical carbon cycle. Geology 1997; 25: 955–6. 10.1130/0091-7613(1997)025<0955:TNFMBA>2.3.CO;2

[71] Hulse D, Ridgwell A. Instability in the geological regulation of Earth’s climate. Science 2025; 389: eadh7730. 10.1126/science.adh7730 40997180

[72] Hilton RG, West AJ. Mountains, erosion and the carbon cycle. Nat Rev Earth Environ 2020; 1: 284–99. 10.1038/s43017-020-0058-6

[73] Lenton TM . The role of land plants, phosphorus weathering and fire in the rise and regulation of atmospheric oxygen. Glob Change Biol 2001; 7: 613–29. 10.1046/j.1354-1013.2001.00429.x

[74] Van Cappellen P, Ingall ED. Redox stabilization of the atmosphere and oceans by phosphorus-limited marine productivity. Science 1996; 271: 493–6. 10.1126/science.271.5248.493 11541251

[75] Longman J, Mills BJW, Merdith AS. Limited long-term cooling effects of Pangaean flood basalt weathering. Nat Commun 2025; 16: 4813. 10.1038/s41467-025-59480-0 40410180 PMC12102204

[76] Caputo MV, Santos ROB. Stratigraphy and ages of four Early Silurian through Late Devonian, early and Middle Mississippian glaciation events in the Parnaíba Basin and adjacent areas, NE Brazil. Earth-Sci Rev 2020; 207: 103002. 10.1016/j.earscirev.2019.103002

[77] Kammer TW, Matchen DL. Evidence for eustasy at the Kinderhookian-Osagean (Mississippian) boundary in the United States: response to late Tournaisian glaciation. In: Fielding CR, Frank TD, Isbell JL (eds.). Resolving the Late Paleozoic Ice Age in Time and Space. Geological Society of America, USA, 2008. 10.1130/978-0-8137-2441-6

[78] Beerling DJ . Low atmospheric CO_2_ levels during the Permo-Carboniferous glaciation inferred from fossil lycopsids. Proc Natl Acad Sci USA 2002; 99: 12567–71. 10.1073/pnas.202304999 12235372 PMC130500

[79] Lowry DP, Poulsen CJ, Horton DE et al. Thresholds for Paleozoic ice sheet initiation. Geology 2014; 42: 627–30. 10.1130/G35615.1

[80] Montañez IP, McElwain JC, Poulsen CJ et al. Climate, *p*CO_2_ and terrestrial carbon cycle linkages during late Palaeozoic glacial–interglacial cycles. Nat Geosci 2016; 9: 824–8. 10.1038/ngeo2822

[81] Saltzman MR, González LA, Lohmann KC. Earliest carboniferous cooling step triggered by the Antler orogeny? Geology 2000; 28: 347–50. 10.1130/0091-7613(2000)28<347:ECCSTB>2.0.CO;2

[82] Donnadieu Y, Goddéris Y, Pierrehumbert R et al. A GEOCLIM simulation of climatic and biogeochemical consequences of Pangea breakup. Geochem Geophys Geosyst 2006; 7: Q11019. 10.1029/2006GC001278

[83] Bergman NM, Lenton TM, Watson AJ. COPSE: a new model of biogeochemical cycling over Phanerozoic time. Am J Sci 2004; 304: 397–437. 10.2475/ajs.304.5.397

[84] Berner RA . GEOCARBSULF: a combined model for Phanerozoic atmospheric O_2_ and CO_2_. Geochim Cosmochim Acta 2006; 70: 5653–64. 10.1016/j.gca.2005.11.032

[85] Rackauckas C, Nie Q. Differentialequations.jl—a performant and feature-rich ecosystem for solving differential equations in Julia. J Open Res Software 2017; 5: 15. 10.5334/jors.151

[86] Bezanson J, Edelman A, Karpinski S et al. Julia: a fresh approach to numerical computing. SIAM Rev 2017; 59: 65–98. 10.1137/141000671

[87] Jacob RL . Low Frequency Variability in a Simulated Atmosphere-Ocean System, PhD. thesis. The University of Wisconsin-Madison, USA, 1997.

