# Supplementary material for: Lithium isotope evidence for enhanced continental weathering triggering the late Paleozoic greenhouse-to-icehouse climate transition
Source: Natl Sci Rev. 2026 Mar 17;13(8):nwag168. doi: 10.1093/nsr/nwag168 (PMC13182253; doi:10.1093/nsr/nwag168)
Supplement: nwag168_Supplemental_File [file nwag168_supplemental_file.docx]

***Supporting Information* for**

**Lithium Isotope Evidence for Enhanced Continental Weathering Triggering the Late Paleozoic Greenhouse-to-Icehouse Climate Transition**

Feifei Zhang^1*,^ Guang-Yi Wei^1^, Pierre Maffre^2^, Ziheng Li^3^, Jianlin Zhou^1^, Alexandre Pohl^4^, Yi-Bo Lin^1^, Maya Elrick^5^, Keyi Cheng^6^, Philip A.E. Pogge von Strandmann^7^‬, Shu-zhong Shen^1^ ‬‬‬‬‬‬‬‬‬‬‬‬‬‬‬‬‬‬‬‬‬‬‬‬‬‬‬‬‬‬‬‬‬‬‬‬‬‬‬‬‬‬‬‬‬‬‬‬‬‬‬‬‬‬‬‬‬‬‬

^1^ State Key Laboratory of Critical Earth Material Cycling and Mineral Deposits, School of Earth Sciences and Engineering, and Frontiers Science Center for Critical Earth Material Cycling, Nanjing University, Nanjing 210023, China

^2^ Aix Marseille Univ, CNRS, IRD, INRAE, CEREGE, Aix-en-Provence, France

^3^ State Key Laboratory of Geomicrobiology and Environmental Changes, China University of Geosciences, Wuhan 430074, China

^4^ Université Bourgogne Europe, CNRS, Biogéosciences UMR 6282, 21000 Dijon, France

^5^ Department of Earth & Planetary Sciences, University of New Mexico, Albuquerque, New Mexico 87131, U.S.A.

^6^ School of Earth and Ocean Sciences, University of Victoria, Victoria, BC, Canada

^7^ MIGHTY (Mainz Isotope and Geochemistry Centre), Institute of Geosciences, Johannes Gutenberg University, 55122 Mainz, Germany

* Corresponding author: Feifei Zhang

Email: fzhang@nju.edu.cn

https://orcid.org/0000-0003-3277-445X

Supplementary Figure. S1 to S13

Supplementary Table. S1 to S3

Supplementary Text

***Stratigraphic correlation***

The stratigraphic correlation between the two sections is primarily based on chronostratigraphic boundaries and carbon isotope trends (Supplementary Fig. 1). The Kinderhookian–Osagean boundary is shown as the black dashed line in both sections, while gray dashed lines mark correlation points based on carbon isotope stratigraphy. According to the six points indicated in the figure, the depth of the **Sacagawea Peak** (SP) section was linearly interpolated to match the depth scale of the **Pahranagat Range** (PR) section (Supplementary Fig. 2). For the post-TICE interval (i.e., above 390.3 m in the SP section), we assume that the sedimentation rate remained constant relative to the overall record. Accordingly, the SP section above the TICE event was proportionally appended above 252 m in the PR section. The total duration of TICE is approximately 4 Myr [1]. We converted the depths of the two profiles into ages and conducted linear interpolation according to the time frame, converting them to ~ -350.9Ma to -346.7Ma [2].

***Elemental and isotopic analyses***

Approximately 400 mg of bulk carbonate powders were dissolved using a well-developed sequential extraction procedure to effectively extract the Li fraction in lattices of carbonate minerals [see details in 3]. After treating with Milli-Q water and 1 M ammonium acetate buffer solution to remove any Li from the water-soluble fraction and exchangeable phases, the samples were dissolved in 1 M acetic acid to extract Li from the carbonate component. The resultant solution was dried down on a hotplate at 100℃. After the complete dry-down, the samples were dissolved and stored in 6 M HCl for elemental and isotopic analysis. An aliquot of each stock solution was dried down and re-dissolved in 3% HNO_3_ for major and trace element analyses. The trace elemental concentrations were determined using a Thermo Element XR™ HR-ICP-MS at the Centre for Research and Education on Biological Evolution and Environment (CREBEE), Nanjing University. An aliquot of solution corresponding to 200 ng Li was taken out from the stock solution, dried down for complete equilibrium, and re-dissolved in 0.2 M HCl for ion-exchange column separation in CREBEE, Nanjing University [3]. A calibrated two columns method with AG50 W-X12 cation resin was used to purify Li following the methods of Pogge von Strandmann *et al.* [4, 5]. The Li yield after the chromatographic steps was > 99% based on the column check on the solution before and after the Li elution interval. Li isotope ratios were measured on a Thermo Neptune XT MC-ICP-MS at the CREBEE, Nanjing University, and the Li isotopes of each sample were normalized to the L-SVEC Li isotope standard. An in-house Li solution (GAGS-Li, δ^7^Li = 15.3‰ ± 0.5‰, 2σ, n = 60 over six months analyses) and OSIL Atlantic seawater (δ^7^Li = 30.7‰ ± 0.4‰, 2σ, n = 15) was used to monitor the long-term reproducibility of Li isotope analyses on the machine. The overall reproducibility and accuracy of the total procedure (sample digestion, Li separation, and isotope measurement) were checked by repeated analyses of carbonate standards GSR-6 (δ^7^Li = –1.75 ± 0.5‰, 2σ, n=4) and GSR-12 (δ^7^Li = 13.5 ± 0.6‰, 2σ, n=4), with different aliquots processed through column chemistry, whose results are consistent with previous studies [3].

***Evidence for primary seawater δ^7^Li values***

We employed a sequential extraction procedure to minimize the influence of detrital components on the δ⁷Li from carbonate fraction. For both sections, the Al/(Mg+Ca) ratios fall below a previously recommended threshold (<0.8 mmol/mol), suggesting limited detrital contamination. However, weak correlations were observed between Al/(Mg+Ca) and δ⁷Li (R² = 0.40, *p* < 0.05 for the PR section, and R² = 0.29, *p* < 0.05 for the SP section, see Supplementary Fig. 5), with a negative correlation in the PR section. To further evaluate the potential detrital impact on δ⁷Li, we calculated the proportion of carbonate-hosted Li in the samples.

$$\begin{aligned} Li_{carb}=Li_{sample}-\left( \frac{Li}{Al} \right)_{UCC}*Al_{sample}\#\left（ 1 \right） \end{aligned}$$

$$\begin{aligned} f_{carb}=1-\left( \frac{Al}{Li} \right)_{sample}*\left( \frac{Li}{Al} \right)_{UCC}\#\left（ 2 \right） \end{aligned}$$

$$\begin{aligned} \delta^{7}Li_{carb}=\left( \delta^{7}Li_{sample}-\delta^{7}Li_{UCC}*\left( 1-f_{carb} \right) \right)/{f_{carb}}\#\left( 3 \right) \end{aligned}$$

Here, $\left( \frac{Li}{Al} \right)_{UCC}$ represents the Li/Al ratio of the upper continental crust, $\left( \frac{Li}{Al} \right)_{UCC}$ = 2.9 × 10⁻⁴, and δ⁷Li_UCC_ is the average Li isotopic composition of the continental crust, set at δ⁷Li_UCC_ = 0.6‰. Using these values, we calculated the proportion of carbonate-hosted Li (f_carb_) for both sections, yielding values over 0.85 for the PR section and 0.94 for the SP section. These results indicate that the detrital contaminations on the Li isotopic signals in both sections are negligible (See Supplementary Fig. 1).

To evaluate the impact on δ^7^Li_carb_ from mineralogy and diagenetic process, we adopted Sr/(Ca+Mg), Mg/(Ca+Mg), Li/(Ca+Mg) and δ^18^O_carb_ to rule out the processes above as the primary drivers of Li isotopic fluctuations in the two sections. We used Mg/(Ca+Mg) and Li/(Ca+Mg) ratios to assess the potential influence of marine diagenesis on the Li isotopic composition of the samples. In the PR section, low Mg/(Ca+Mg) (0.01–0.05) and Sr/(Ca+Mg) (0.3–1.3) values indicate marine diagenesis. In the SP section, Sr/(Ca+Mg) values similarly remain low (0.03–1.5), while some Mg/(Ca+Mg) ratios are notably higher (0.01–0.48). These elevated Mg ratios are mostly observed in the upper part of the SP section after the TICE event, where δ⁷Li values and Li concentrations are also relatively high, suggesting that these samples may have experienced fluid-buffered dolomitization [3].

In the PR section, δ⁷Li exhibits weak correlations with both Sr/(Ca+Mg) (R^2^ = 0.31, *p* <0.05) and Li/(Ca+Mg) (R^2^ = 0.18, *p* <0.05). However, these ratios show weaker correlations with δ¹⁸O during the TICE interval (samples above H = 66 m in the PR section, R^2^ = 0.22, *p* <0.05 and R^2^ = 0.23, *p* <0.05). This suggests that the Li isotopic excursion observed during the TICE event is unlikely to have been driven by changes in carbonate mineralogy or diagenesis.

***GEOCLIM model***

We used the version 7.0 of GEOCLIM published in Maffre *et al.*[6], with several modifications. The computation of erosion rates (*E*) was updated to represent an uplift by adding the factors *f(t)* and *g(x,y)* to the erosion equation: *E = k_e_*q^0.5^*s*(1 + f(t)*g(x,y))*. *f(t)* represents the temporal evolution of the uplift pulse. We chose a lognormal functional form, as indicated in the main text: *f(t) = (A_p_-1) * exp(-σ_p_^2^/2) * (t_p_*exp(σ_p_^2^)/t) * exp[-log(t/(t_p_*exp(σ_p_^2^))**2 / (2*σ_p_^2^)]*, where *A_p_* is the amplitude of the pulse (= 20), *t_p_* is the timing of the peak (= 3 Myr) and *σ_p_* (= 0.3) determines the temporal width of the pulse. *g(x,y)* is a geographic mask: 1 for the uplifted areas, 0 for the others. In our simulation, the uplift is located on the western part of Gondwana (see Supplementary Fig. 3 B0-1).

The other main update is the addition of lithium isotopic cycle. For the continental component, we used the model of Caves Rugenstein *et al.* [7] to compute both the lithium flux from silicate weathering (*F^wth^_Li_*) and the associated isotopic signature δ^7^Li_riv_. The first step is to estimate the Total Dissolved Solid flux (TDS) from silicate weathering: *TDS_sil_ = (500/2500)*F^wth^_sil_*, where is the Ca-Mg flux from silicate weathering (computed by GEOCLIM) and 500/2500 is the ratio of modern world-average estimates of TDS_sil_ (in 10^9^ kg/yr) and Ca-Mg flux (in 10^9^ mol/yr) coming from Gaillardet *et al.* [8]. The weathering intensity (*WI*) is then computed as *TDS_sil_/F_E_,* with *F_E_* the erosion flux computed by GEOCLIM, in kg/yr. Finally, the equations from Caves Rugenstein *et al.* [7] are:

*δ^7^Li_riv_ = δ^7^Li_ucc_ + ((1 - WI)/WI) * a * exp(-b/WI)*

*F^wth^_Li_ = F^wth^_sil_ * ([Li]/[CaMg]) * exp((*δ^7^Li_riv_ – δ^7^Li_ucc_*) / Δ_sec_)*

The parameters of this model are *a* (= 4.5‰), *b* (= 0.0575), the fractionation coefficient during incorporation in secondary phases *Δ_sec_* (= -17‰), the Li concentration in bedrock *[Li]* (= 0.011 mol/kg), and the Li isotopic ratio in berock *δ^7^Li_ucc_* (= 1.7‰). The concentration of Ca-Mg in bedrock *[CaMg],* already defined in GEOCLIM, is only used to scale the Li flux to the silicate weathering flux.

For the oceanic component, we assumed a constant hydrothermal flux of 13 Gmol/yr with an isotopic composition δ^7^Li_hyd_ = 8.3‰ [9], and a unique Li sink, the incorporation in authigenic clay *F^auth^_Li_*, set proportional to the oceanic Li concentration: *F^auth^_Li_ = k^auth^_Li_*[Li]*V*, where *V* is the ocean volume, and *k^auth^_Li_* is a proportionality constant. This Li sink has a fractionation coefficient *Δ_sink_* = -15‰ for modern ocean [9]. The mass and isotopic balance equations used to compute the dynamic evolution of Li cycle are identical to Eq. 4 and 5 of next section (“COPSE model”).

Forcing fields of surface air temperature and runoff needed in GEOCLIM were provided by new ocean-atmosphere general circulation model simulations conducted using the Fast Ocean-Atmosphere Model (FOAM) v1.5 (ref. [10]) for atmospheric CO_2_ concentrations of 140 ppm, 280 ppm, 560 ppm, 1120 ppm, 2240 ppm, 4480 ppm and 8960 ppm, using the 360 Ma paleogeographical configuration of Scotese and Wright [11], a null-eccentricity-minimum obliquity orbital configuration and a solar luminosity value reduced to 1327.33 W m^–2^ calculated for 360 Ma after Gough [12]. We conducted a first GEOCLIM simulation to calibrate the model with FOAM climate outputs from a pre-industrial run. This step was necessary since Maffre *et al.* [6] calibration was done with climate outputs from an IPSL-CM5A2 simulation, while in this study, we used FOAM to compute Tournaisian climate. In this pre-industrial calibration, we slightly altered the rate constants for PIC dissolution below the lysocline, POC remineralization in sediments by O_2_ and by sulfate-reduction, in order to achieve pre-industrial lysocline depth, O_2_ and SO_4_^2-^ concentrations using FOAM outputs. These parameters were thereafter kept identical for Tournaisian simulations. The proportionality constant *k^auth^_Li_* was tuned in order to get the steady-state of Li cycle for modern Li oceanic concentration .

Some other modifications were made for the Tournaisian simulation. First, the presence of pelagic carbonate producers was disabled to represent Paleozoic oceans (the made-up flag in the code was switched off) [13].We explicitly represented the paleo-Tethys in GEOCLIM, in a separate “column of boxes”, in order to capture the anoxic potential of this partially isolated basin. We used the configuration where one coastal box column is defined for each open-ocean basin, instead of the default single coastal box column [6]. The seafloor depth threshold for the definition of coastal boxes was set to 250m to match FOAM bathymetry that only has discrete values of 100 m and 250 m. The continental drainage basins were determined by the closest oceanic point method, and modified so that the water divides follow the middle of the main mountain ranges (see Supplementary Fig. 3A). Although Caves Rugenstein *et al.* [7] continental lithium model was designed to represent to Earth-integrated Li flux and isotopic ratio, to stay consistent with the routing of continental fluxes into GEOCLIM boxes, we applied this model to each drainage area, which is still consistent since they all are continental-wide. Because no deep-water formation occurs in Northern hemisphere in FOAM simulations, we assigned 3 vertical levels in GEOCLIM Northern high latitude (i.e., > 60°N) box column. We designed the southern high latitude box to capture the deep water formation on the South-West Gondwana margin, keeping the 2 standard vertical levels. The temperature and water exchanges between oceanic boxes were determined from FOAM simulations with the method presented in Maffre *et al.* [6]. However, we used constant (i.e., *p*CO_2_-invariant) water flux from the 2240 ppmv simulation, because FOAM simulations at lower pCO_2_ exhibit a climatic instability [14] whose existence may be model-dependent. Because of the exceptionally large shallow water areas in the paleogeographic reconstruction, the sediment accumulation capacity had to be modified to avoid an unrealistic behavior where virtually all of the sedimentary flux is located in the shallowest boxes. We divided the accumulation capacity by 30 in the coastal surface boxes, and by 2.5 in the coastal boxes below 100m. In the absence of any information on continental lithology in Tournaisian age, we put a uniform lithology on lands and used the seawater ^87^Sr/^86^Sr ratio as constraint. The following lithological assemblage was designed to fit the Tournaisian seawater ^87^Sr/^86^Sr ratio of 0.7083 before the erosion perturbation 10% metamorphic, 17%, felsic 2% intermediate, 12% mafic, 50% siliclastic sediment, and 9% carbonate. The slope field was reconstructed following the method of Maffre *et al.* [6], and increased so that the *WI* is ~0.05, corresponding to the maximum δ^7^Li_riv_ in Caves Rugenstein *et al.* [7]. Finally, the magmatic CO_2_ degassing was tuned to 11 Tmol(C)/yr (~ 3 times pre-industrial), so that the pre-perturbation *p*CO_2_ corresponds to equatorial SST (Supplementary Fig. 4C) matching the TICE estimate of ~30°C (ref. [15]). The lithium cycle in late Paleozoic has notable differences with the modern cycle because of the elevated rate of reverse weathering before the rise of diatoms in global ocean [16, 17]. This implies that the residence time of Li in seawater must be lower (more efficient Li sink) and that the fractionation of the Li sink (the isotopic offsets between authigenic clays and seawater, *Δ_sink_*) is also lower. We set the value of *Δ_sink_* to -5‰, a value proposed for Late Paleozoic [16] and in the absence of any better constraint, we assumed a three-fold reduction of this residence time, to ~0.5 Myr by dividing *k^auth^_Li_* by 3. We note that GEOCLIM does not take into account the sink of major cations and alkalinity due to reverse weathering. We conducted a sensitivity test to represent these sinks and concluded that it does not affect our results and interpretations (see section “*GEOCLIM extended model runs*”). In Fig. 4G, we present a sensitivity test where *Δ_sink_* is set to -7‰. Rather than re-running GEOCLIM, we simply solved the mean *δ^7^Li_sw_* differential equation (Eq. 5) with this new *Δ_sink_*, using the time-series of Li fluxes and concentration from the main GEOCLIM run. We note that the “full” GEOCLIM simulation was actually the one ran with a *Δ_sink_* of -7‰, and the “offline solved” *δ^7^Li_sw_* equation the one with a *Δ_sink_* of -5‰, but this has no influence on the interpretation, both being accurate computation of the *δ^7^Li_sw_* evolution.

***GEOCLIM extended model runs***

In addition to the *Δ_sink_* sensitivity, we ran two other series of sensitivity test. The first one consists in explicitly modelling the sink of major cations (Ca^2+^ and Mg^2+^) and akalinity by reverse weathering. In the absence of any further information, we assumed that a factor *f_rw_* of the cations and alkinity flux to the ocean from silicate weathering is immediately consumed by reverse weathering. To keep a similar initial condition in term of global mean temperature, the background magmatic CO_2_ degassing was reduced by *f_rw_* (i.e., multiplied by “1-*f_rw_*”). Two values were tested for *f_rw_*: 0.5 and 0.75. To stay consistent, we also reduced the hydrothermal Li input by *f_rw_*. The results of these two simulations are shown in Supplementary Fig. S10 and S11. They do not exhibit any significant difference with the “main” simulation. The residence time of C is increased because of the reduced source of C, therefore, with higher *f_rw_*, the time of recovery after the perturbation is longer. The initial δ^7^Li_sw_ is more elevated (up to +3.2‰) due to the reduced hydrothermal input, but the lowest value reached during the excursion is virtually identical in all cases. The δ^13^C is shifted by up to +0.4‰, because lowering the CO_2_ degassing changes the balance between the isotopically distinct C sources: magmatic degassing, carbonate weathering and fossil organic C weathering.

The second sensitivity test concerns a source of uncertainty regarding the paleogeography. Updated paleogeographic reconstructions [18, 19] display less inundated shelves and more exposed lands than the one we use, which is arguably the most significant difference. To evaluate this impact, we modified our simulation by reducing the area of oceanic boxes shallower than 250m (area multiplied by 0.25, see Supplementary Fig. S12) and the increasing the area of land pixels lower than 250 m (area multiplied 2.679, see Supplementary Fig S12). The ratio of 0.25 was arbitrarily chosen to represent a large reduction of inundated shelves, and the ratio of 2.679 is the one that verifies the conservation the total Earth area when applied to all land pixels lower than 250m. Because of the increased total land area, the magmatic CO_2_ degassing had to be raised to 15 Tmol/yr to compensate higher silicate weathering flux and keep an similar initial climate. In response to the uplift perturbation, this new simulation (Supplementary Fig. S13) exhibits very little difference in term of δ^7^Li, although the absolute fluxes of erosion and weathering are higher. The main differences arises from the reduced extent of inundated shelf, and potentially because of the higher global O_2_ level (linked to the higher global weathering): the response of organic C burial is somewhat muted, the δ^13^C excursion is reduced, as well as the global cooling, to a lesser extent. This sensitivity test demonstrates that our results are robust to uncertainties in the position of the paleo-coastlines. It further suggests that a large fraction of inundated shelves is likely needed to explain the amplitude of the TICE.

***COPSE model***

**Summary.** The model is a reloaded version of the COPSE with extended lithium cycle [20, 21]. The biogeochemical Earth system model represents the long timescale (≥ 10^5^‒10^8^yr) evolution of the global atmosphere-ocean carbon (C), oxygen (O), ocean phosphorus (P), ocean sulphur (S), ocean nitrogen (N) and lithium (Li) as a set of coupled ordinary diﬀerential equations [20, their Table 1]. The model is perturbed by forcing parameters vegetation (V) and degassing (D); additional riverine lithium input fluxes; and the isotopic values of the riverine lithium, then calculates the response of the C-Li cycles.

Briefly, silicate weathering responds to the changes in V which acts as the sink of carbon through carbonate burial, and the source of Li to the ocean. Organic carbon and carbonate burial acts as sink of carbon. Degassing responds to the changes in D which acts as the source of carbon and oceanic Li.

**Lithium cycle.** The behavior of Li and its isotopes was modelled using the equations from [4, 21]. A 4‰ offset between the lithium isotope signal from original seawater and carbonates was considered here. The models were constructed from the mass balance equation:

$$\begin{aligned} \frac{dLi}{dt}=F_{riv}+F_{h}-F_{sed}\#\left( 4 \right) \end{aligned}$$

where **Li** is the seawater Li reservoir (in mol), and F_x_ represents the input and output fluxes (in mol/yr) (riv = river, h=hydrothermal, sed=sediment (combined low temperature basalt alteration and sediment diagenesis)). The isotopic balance equation is then given by:

$$\begin{aligned} Li\frac{d\delta^{7}Li_{sw}}{dt}=F_{riv}\left( \delta^{7}Li_{riv}-\delta^{7}Li_{sw} \right)+F_{h}\left( \delta^{7}Li_{h}-\delta^{7}Li_{sw} \right)-F_{sed}*\triangle_{sink}\#\left( 5 \right) \end{aligned}$$

where δ^7^Li_x_ (sw = seawater) is the isotope ratio of the fluxes. δ^7^Li_h_ varying between 0 to 25‰ as tested below, δ^7^Li_h_ = 7‰, Δ_sink_ = δ^7^Li_sed_ - δ^7^Li_sw_ = -5‰. In this model, lithium cycle is coupled to carbon cycle:

$$\begin{aligned} F_{h}=k_{h}*D\#\left( 6 \right) \end{aligned}$$

where k_h_ = 1.2e10 (mol/yr), the hydrothermal input flux of lithium, F_h_, is taken to scale with sea floor spreading rate and therefore degassing.

$$\begin{aligned} F_{r}=\frac{k_{r}*F_{silw}}{k_{silw}}*k_{1}\#\left( 7 \right) \end{aligned}$$

where k_r_ = 1.7e10 (mol/yr), the riverine flux of lithium, F_r_, is assumed proportional to the silicate weathering flux. Hence, in the initial steady state oceanic lithium yields a ~1 Myr residence time (**Li** = 3.5e16 mol, total input flux = 2.9e10 mol/yr). We also include an optional multipler k_1_, which adjust the riverine lithium flux relative to the silicate inputs to account for Li partitioned from the bedrock into the dissolved load under various weathering regimes (*5*).

$$\begin{aligned} F_{sed}=k_{sed}*Li\#\left( 8 \right) \end{aligned}$$

the sink of Li is assumed proportional to the oceanic Li reservoir with a coefficient constant k_sed_ = 2.9e10 (mol/yr).

***COPSE extended model runs and sensitivity tests***

There are three groups of model runs employed to do the sensitive tests of: the forcing vegetation (V), the forcing degassing (D), the extra riverine lithium input controlled by multiplyer k_1_, the isotopic value of the riverine lithium input.

The 1^st^ group of extended model runs (Supplementary Fig.5) focuses on modifying the forcing V by using the following functions with other three key parameters (D = 1.6, k_1_ = 1.0, δ^7^Li_riv_ = 18‰) unchanged:

V = [-1e30, -350.2e6, -349.7e6, -349.0e6, -348.4e6, -348e6, -347e6, 1e30],

[Vbaseline, Vbaseline, Vpeak1, Vpeak1, Vpeak2, Vpeak2, Vpeak1, 1.0]

Here the first vector is the time in years, and the second shows forcing (or flux) multiplier or values at these times. Vpeak1 (= 0.5, 0.9, 1.2), Vpeak1 (= 1.0, 1.6, 2.0) and Vpeak2 (= 1.0, 2.0, 2.2) are the values of forcing V. Basically we increase the vegetation to near present level, hence, increase the organic carbon burial on land. The organic carbon burial is the source of atmosphere oxygen, and the sink of atmosphere-ocean carbon. As shown in Supplementary Fig.6, the increasing V results in the positive excursion in carbonate carbon isotopes, the drop of pCO_2_ and limits the silicate weathering. However, the changes in the silicate weathering cannot result in the great negative lithium isotope excursion as we observed.

The 2^nd^ group of extended model runs (Supplementary Fig.7) inherit the V pattern from the major model run in the main text, focusing on testing the forcing D by using the following functions and fix the other two parameters (k_1_ = 1.0, δ^7^Li_riv_ = 18‰):

D = [-1e30, -350e6-1, -350e6, -347e6, 1e30],

[1.6, 1.6, Dpeak, Dpeak, 1.6]

Here the first vector is the time in years, and the second shows forcing (or flux) multiplier or values at these times. Dpeak (= 1.0, 3.0, 5.0) are the values of forcing D. We can see in the Supplementary Fig.7 that D accelerates with higher pCO_2_, higher carbon burial and riverine lithium input. However, as revealed in previous studies the degassing drove hydrothermal input in isolation is not a significant driver of the δ^7^Li composition of seawater [21].

The 3^rd^ group of extended model runs (Supplementary Fig.8) inherit the V pattern from the major model run in the main text. Focusing on testing the isotopic values of the riverine lithium input by using the following functions with other parameters fixed (D = 1.6, k1 = 1.0):

δ^7^Li_riv_ = [-1e30, -351e6, -350.2e6, -349.5e6, -349.5e6+1, -348.4e6, -348.4e6+1, -348e6, -347.5e6, 1e30l, [23.0,23.0, Lipeak1, Lipeak1, Lipeak2, Lipeak2, Lipeak1, Lipeak1, Lipeak2, Lipeak2]

Here the first vector is the time in years, and the second shows forcing (or flux) multiplier or values at these times. Lipeak1 (= 0.0, 0.0, 5.0), Lipeak2 (= 10, 18, 25) are the values of δ^7^Li_riv_. Given the δ^7^Li_riv_ varies dramatically with different weathering intensity [22], the changes in δ^7^Li_riv_ might play key role in leading to the negative δ^7^Li_sw_ excursion as we observed. We can see in the Supplementary Fig.8 that without change in the riverine lithium input flux, the changes in δ^7^Li_riv_ in isolation can lead to a -6‰ δ^7^Li_sw_ excursion, nearly half of the magnitude of first (at 349.5 Ma) negative δ^7^Li_sw_ peak.

The 4^th^ group of extended model runs (Supplementary Fig.9) inherit the V, D and δ^7^Li_riv_ patterns from the major model run in the main text. Focusing on testing the flux of the riverine lithium input by using the following functions:

k_1_ = [-1e30, -351e6, -350.2e6, -349.5e6, -349.5e6+1, -348.4e6, -348.4e6+1, -348e6, -347.5e6, 1e30], [1.0, 1.0, kpeak1, kpeak1, kpeak2, kpeak2, kpeak3, kpeak3, kpeak3, kpeak3]

Here the first vector is the time in years, and the second shows forcing (or flux) multiplier or values at these times. kpeak1 = (2.0, 5.0, 7.0), kpeak2 = (0.5, 1.5, 2.5), kpeak3 = (3.0, 6.0, 8.0). The multiplier is used to model the uncertainty that the riverine lithium input may be enhanced by the enhancement of the silicate weathering flux, and the magnitude of the effect is not simply linearly correlated. As expect, the model prediction matches up well with the data when both changes in flux and isotopic values of the riverine lithium input are considered.


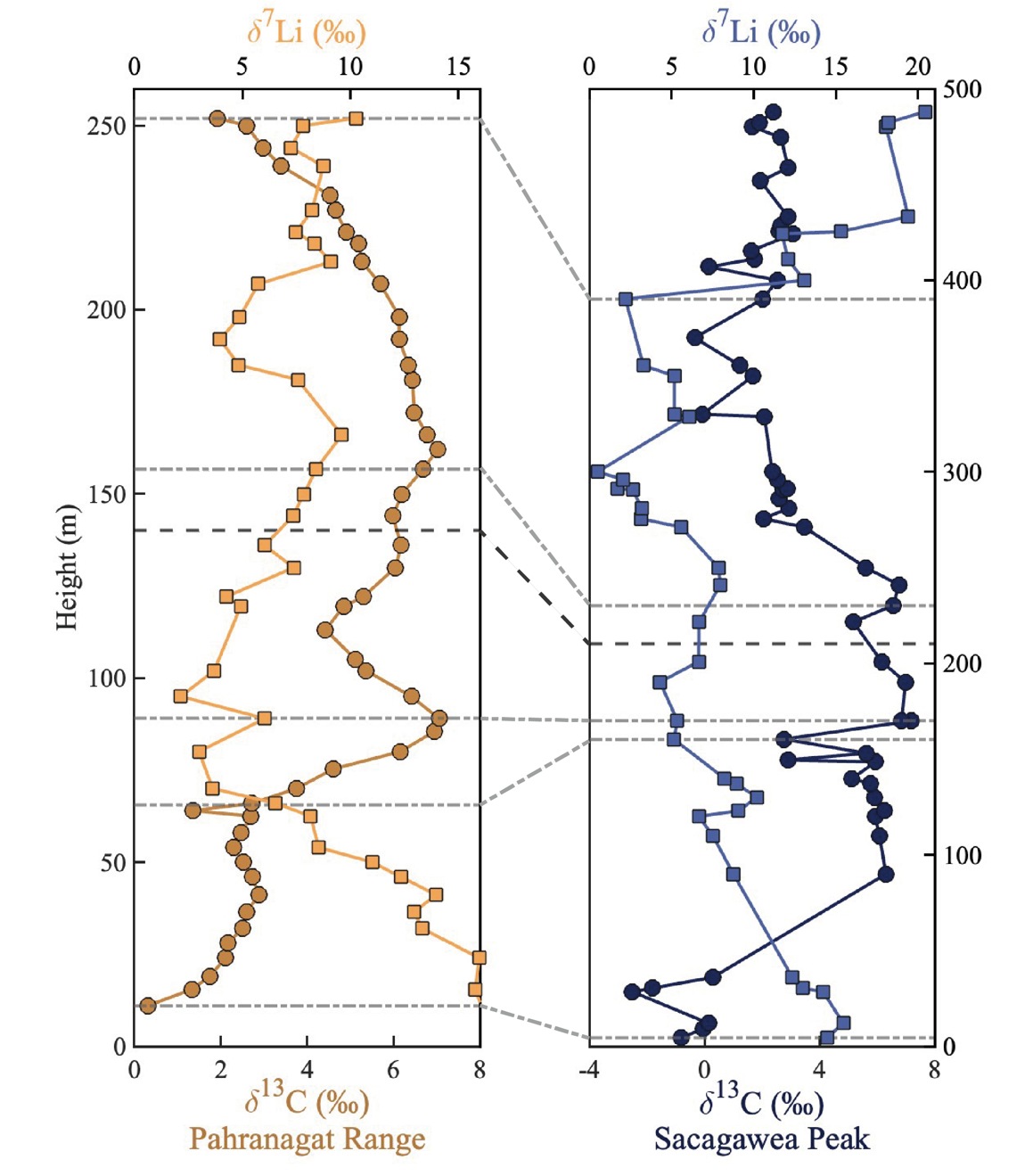


Supplementary Figure S1. Stratigraphic correlation between δ^13^C_carb_ and δ^7^Li from Pahranagat Range and Sacagawea Peak sections. Dots refer to δ^13^C_carb,_ while squares refer to δ^7^Li. The black marks the Kinderhookian-Osagean boundary.


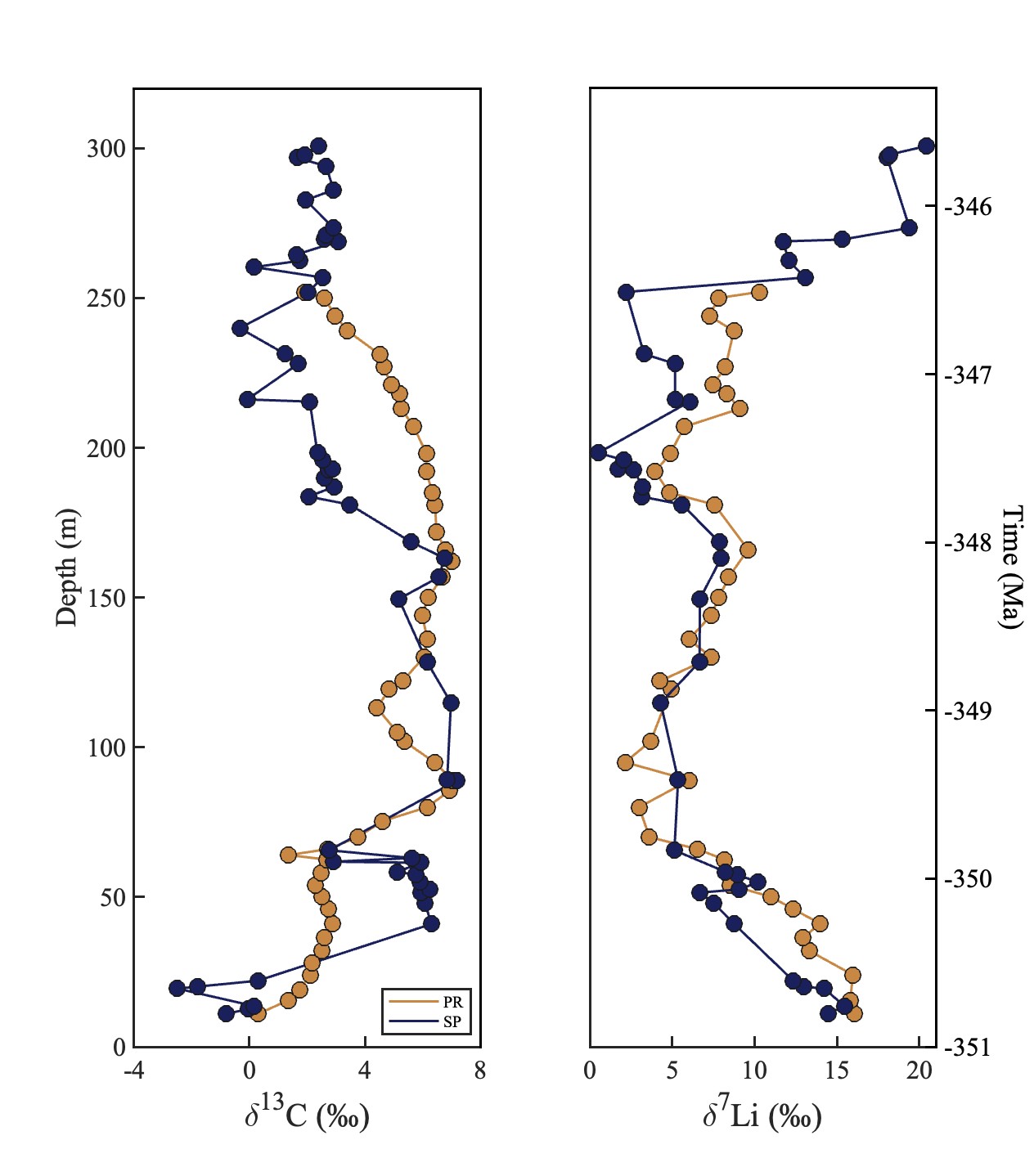


Supplementary Figure S2. δ^13^C_carb_ and δ^7^Li in Pahranagat Range and Sacagawea Peak sections, with depth and time scale transformed to the scale of Pahranagat Range section. Dots refer to δ^13^C_carb,_ while squares refer to δ^7^Li.


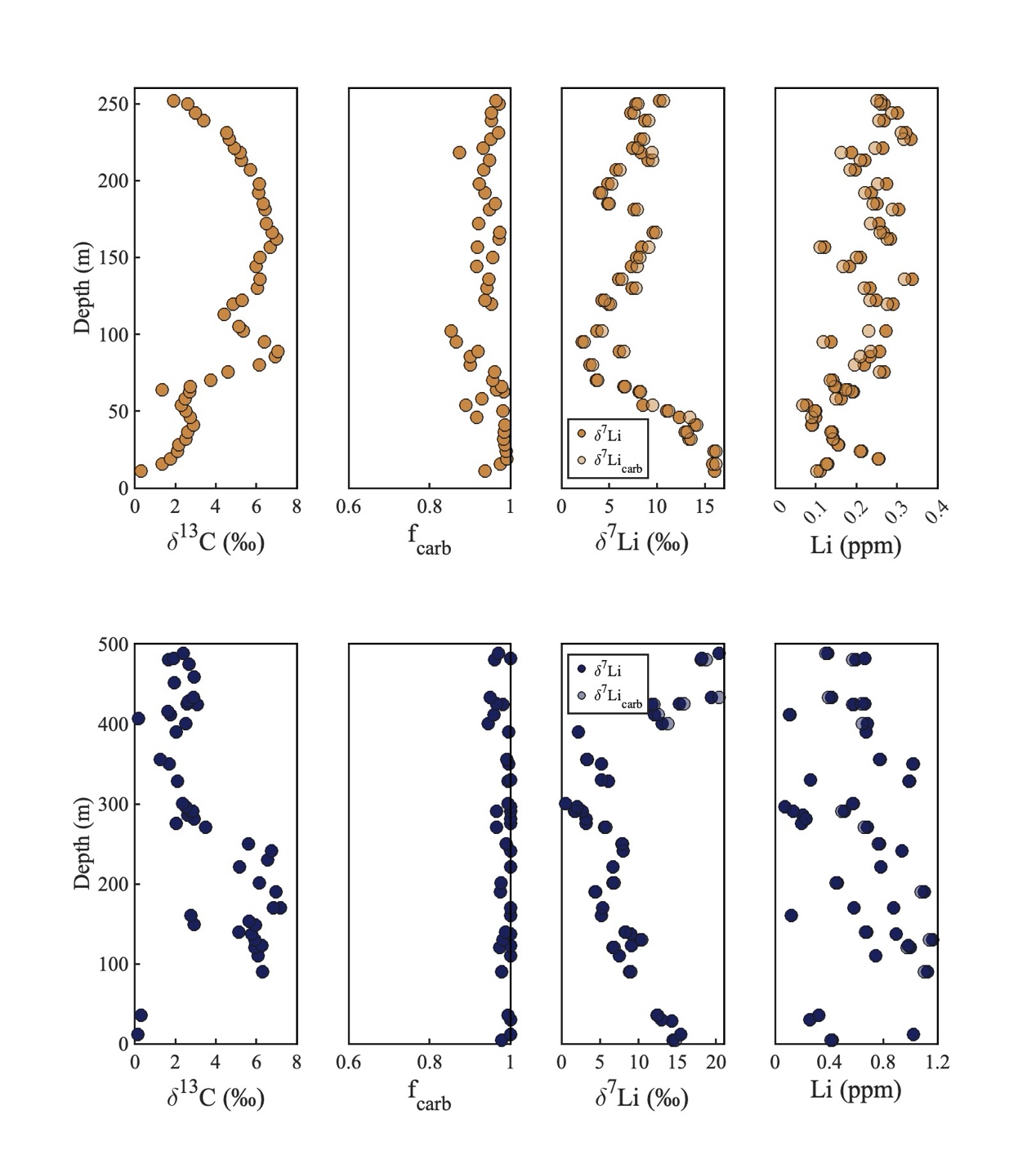


Supplementary Figure S3. δ^13^C_carb_, δ^7^Li, δ^7^Li_carb,_ Li and Li_carb_ content results for Pahranagat Range and Sacagawea Peak.


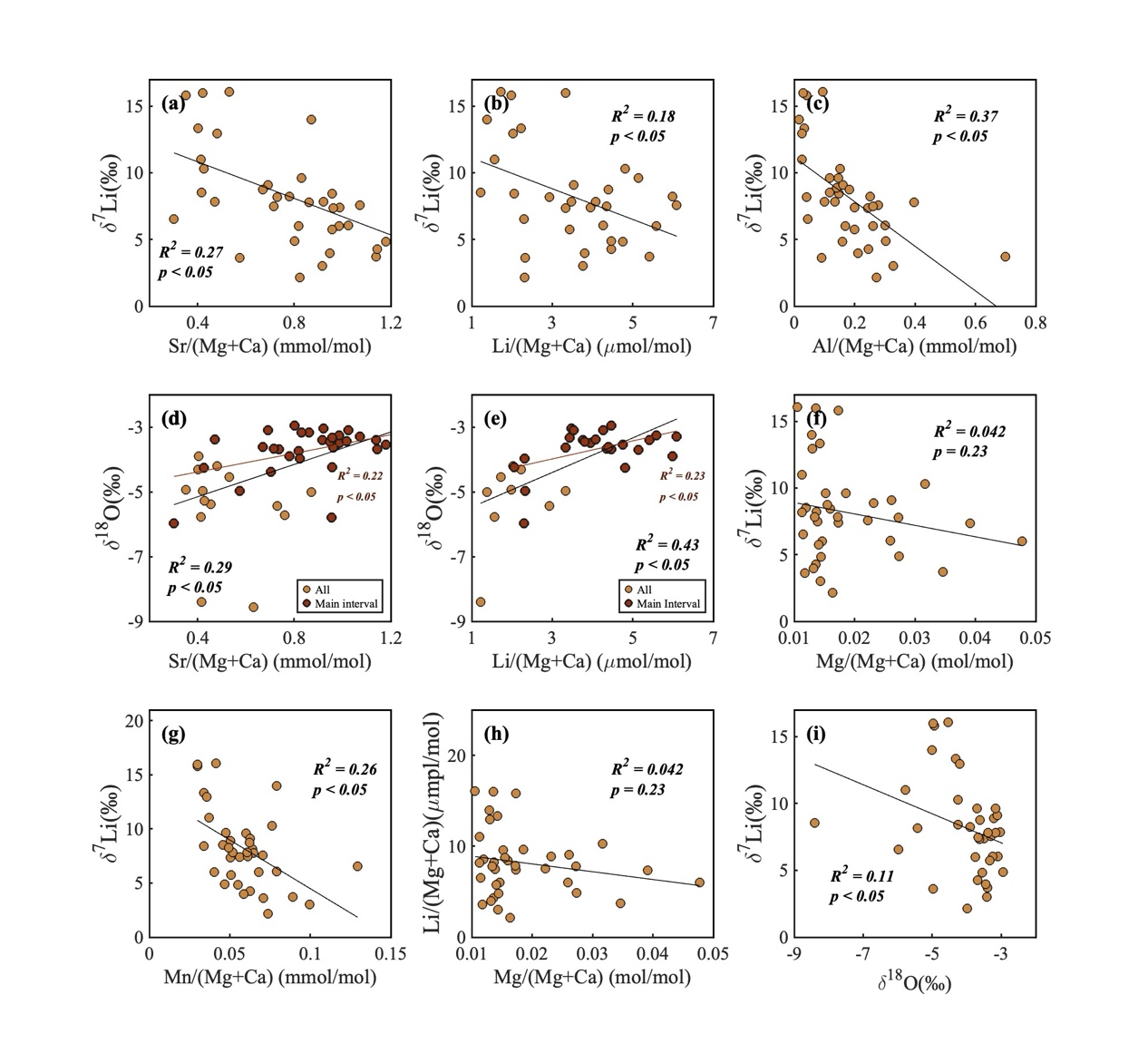


Supplementary Figure S4. Cross-plots of Pahranagat Range section. (a) δ^7^Li versus Sr/(Mg+Ca). (b) δ^7^Li versus Li/(Mg+Ca). (c) δ^7^Li versus Al/(Mg+Ca). (d) δ^18^O versus Sr/(Mg+Ca). Dark red dots refer to data during main interval (Height >= 66m). (e) δ^18^O versus Li/(Mg+Ca). (f) δ^7^Li versus Mg/(Mg+Ca). (g) δ^7^Li versus Mn/(Mg+Ca). (h) Li/(Mg+Ca) versus Mg/(Mg+Ca). (i) δ^7^Li versus δ^13^C.


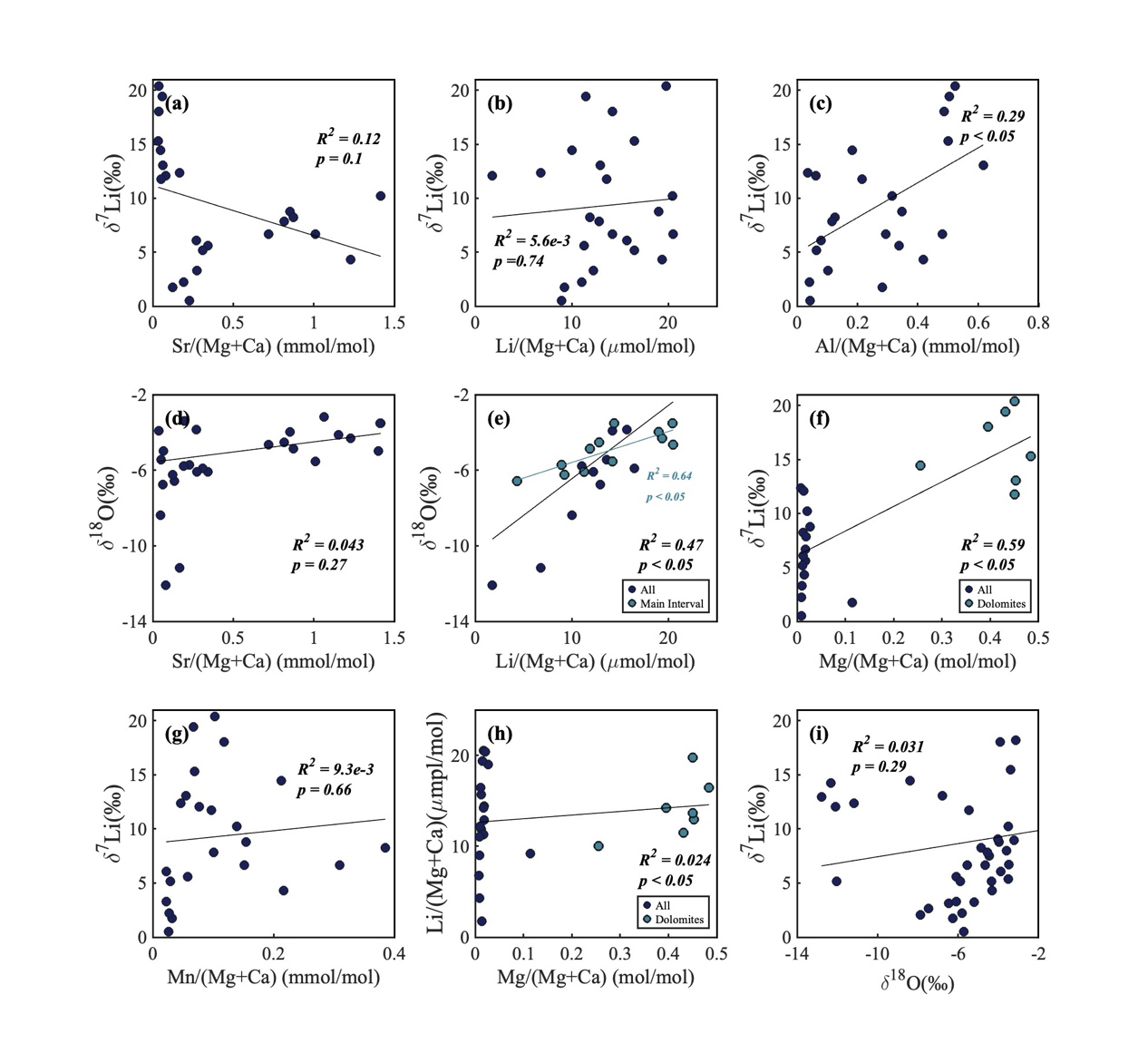


Supplementary Figure S5. Cross-plots of Sacagawea Peak section. （a）δ^7^Li versus Sr/(Mg+Ca). (b) δ^7^Li versus Li/(Mg+Ca). (c) δ^7^Li versus Al/(Mg+Ca). (d) δ^18^O versus Sr/(Mg+Ca). Light blue dots refer to data during main interval (50m <Height <= 300m). (e) δ^18^O versus Li/(Mg+Ca). (f) δ^7^Li versus Mg/(Mg+Ca). (g) δ^7^Li versus Mn/(Mg+Ca). (h) Li/(Mg+Ca) versus Mg/(Mg+Ca). (i) δ^7^Li versus δ^13^C.


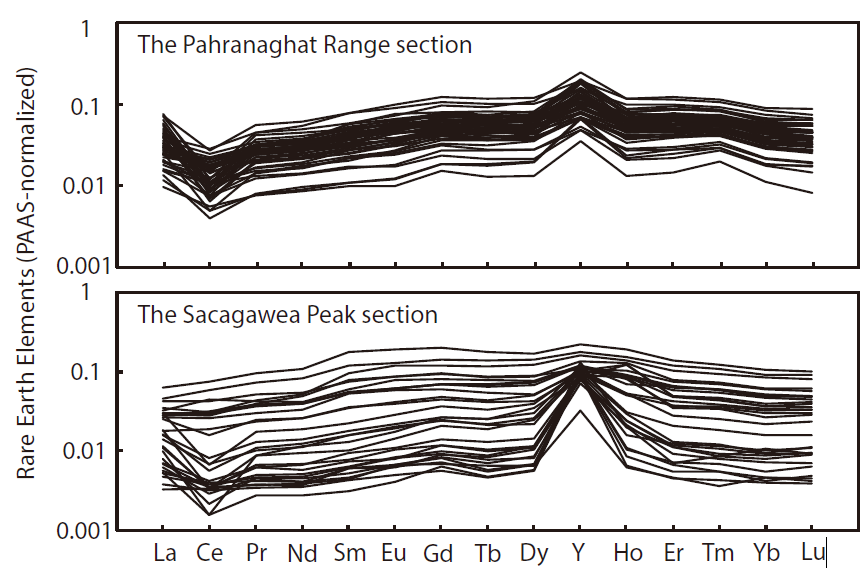


**Supplementary Figure S6. The rare earth element (REE) patterns of the Sacagawea Peak section and the Sacagawea Peak section.** The contents of REEs are normalized by Post-Archean Australia Shale (PAAS)[23].


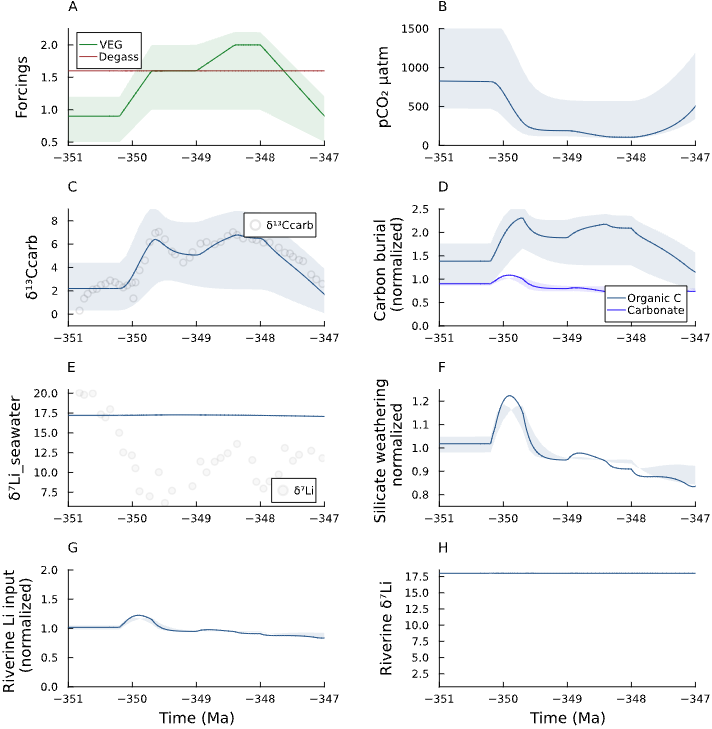


Supplementary Figure S6. Sensitivity tests of the forcing vegetation (V). (A) COPSE forcings Vegetation (V, green line, green bar shows the sensitivity tests) and Degassing (D, orange line and bar). (B) pCO_2_ (μatm). (C) Model-data comparison of δ^13^C_carb_. (D) Normalized marine carbonate (dark blue) and organic carbon (blue) burial flux. (E) Model-data comparison of δ^7^Li. (F) Normalized silicate weathering flux. (G) Normalized riverine lithium input flux. (H) Isotopic values of the riverine lithium input.


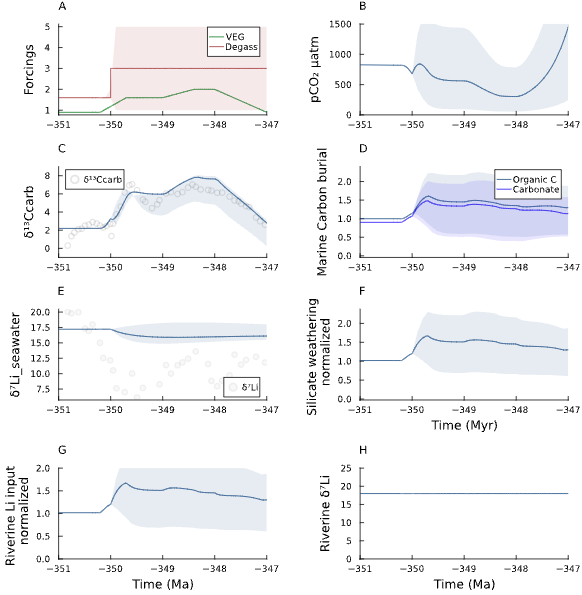


Supplementary Figure S7. Sensitivity tests of the forcing degassing (D). (A) COPSE forcings Vegetation (V, green line) and Degassing (D, orange line, brown bar shows the sensitivity tests). (B) *p*CO_2_ (ppmv). (C) Model-data comparison of δ^13^C_carb_. (D) Normalized marine carbonate (dark blue) and organic carbon (blue) burial flux. (E) Model-data comparison of δ^7^Li. (F) Normalized silicate weathering flux. (G) Normalized riverine lithium input flux. (H) Isotopic values of the riverine lithium input.


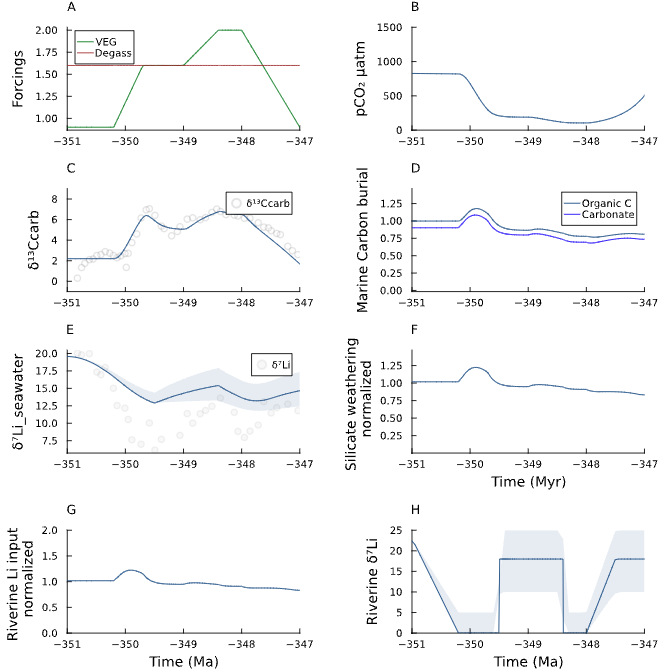


Supplementary Figure S8. Sensitivity tests of the δ^7^Li_riv_. (A) COPSE forcings Vegetation (V, green line) and Degassing (D, orange line). (B) pCO_2_ (μatm). (C) Model-data comparison of δ^13^C_carb_. (D) Normalized marine carbonate (dark blue) and organic carbon (blue) burial flux. (E) Model-data comparison of δ^7^Li. (F) Normalized silicate weathering flux. (G) Normalized riverine lithium input flux. (H) Isotopic values of the riverine lithium input, the blue bar shows the sensitivity tests.


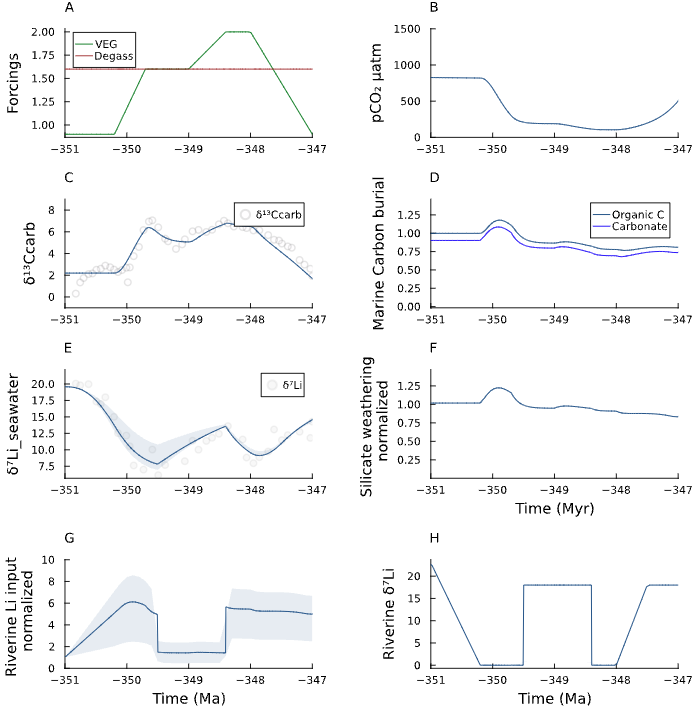


Supplementary Figure S9. Sensitivity tests of the δ^7^Li_riv_. (A) COPSE forcings Vegetation (V, green line) and Degassing (D, orange line). (B) *p*CO_2_ (ppmv). (C) Model-data comparison of δ^13^C_carb_. (D) Normalized marine carbonate (dark blue) and organic carbon (blue) burial flux. (E) Model-data comparison of δ^7^Li. (F) Normalized silicate weathering flux. (G) Normalized riverine lithium input flux, the blue bar shows the sensitivity tests. (H) Isotopic values of the riverine lithium input.


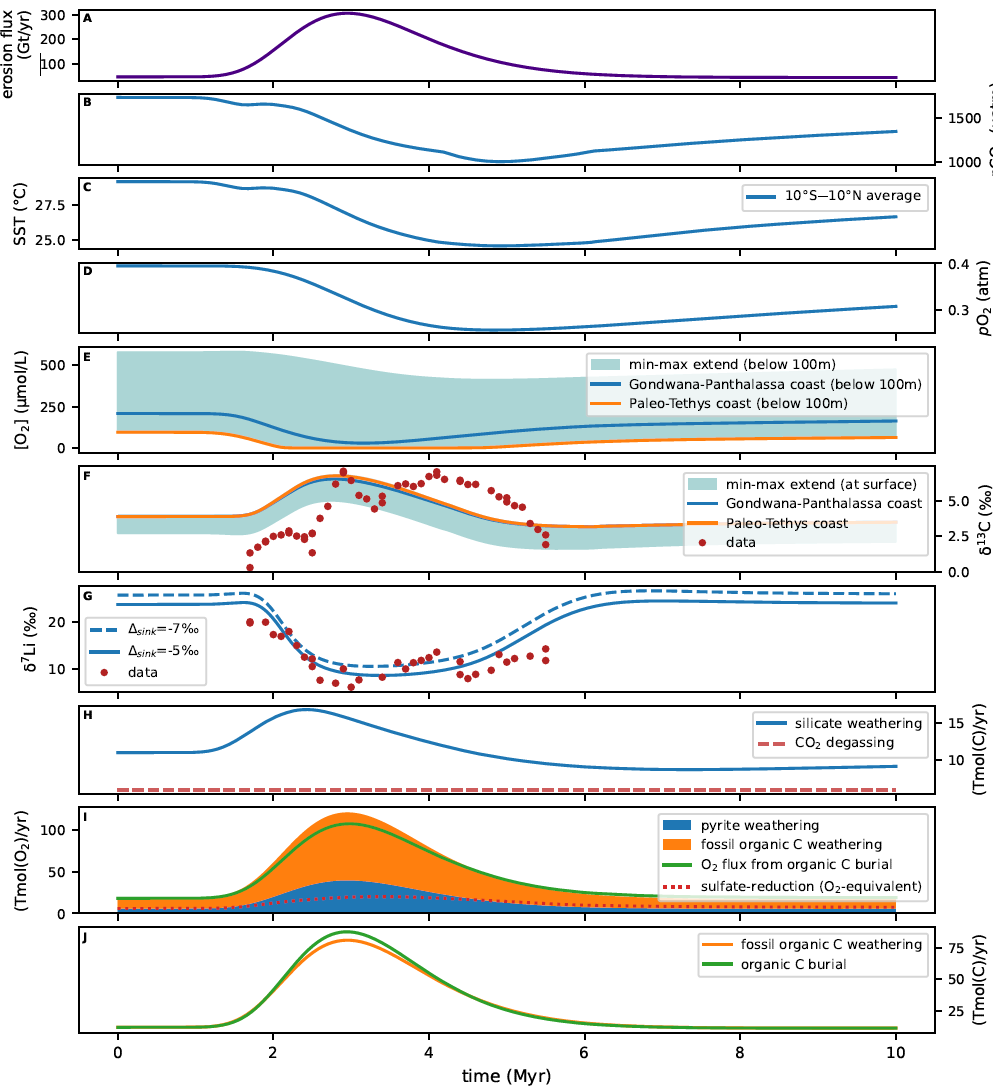


**Supplementary Figure S10.** Same than main text Fig. 4 for the GEOCLIM simulation with 50% of “explicit” reverse weathering (*f_rw_*=0.5).


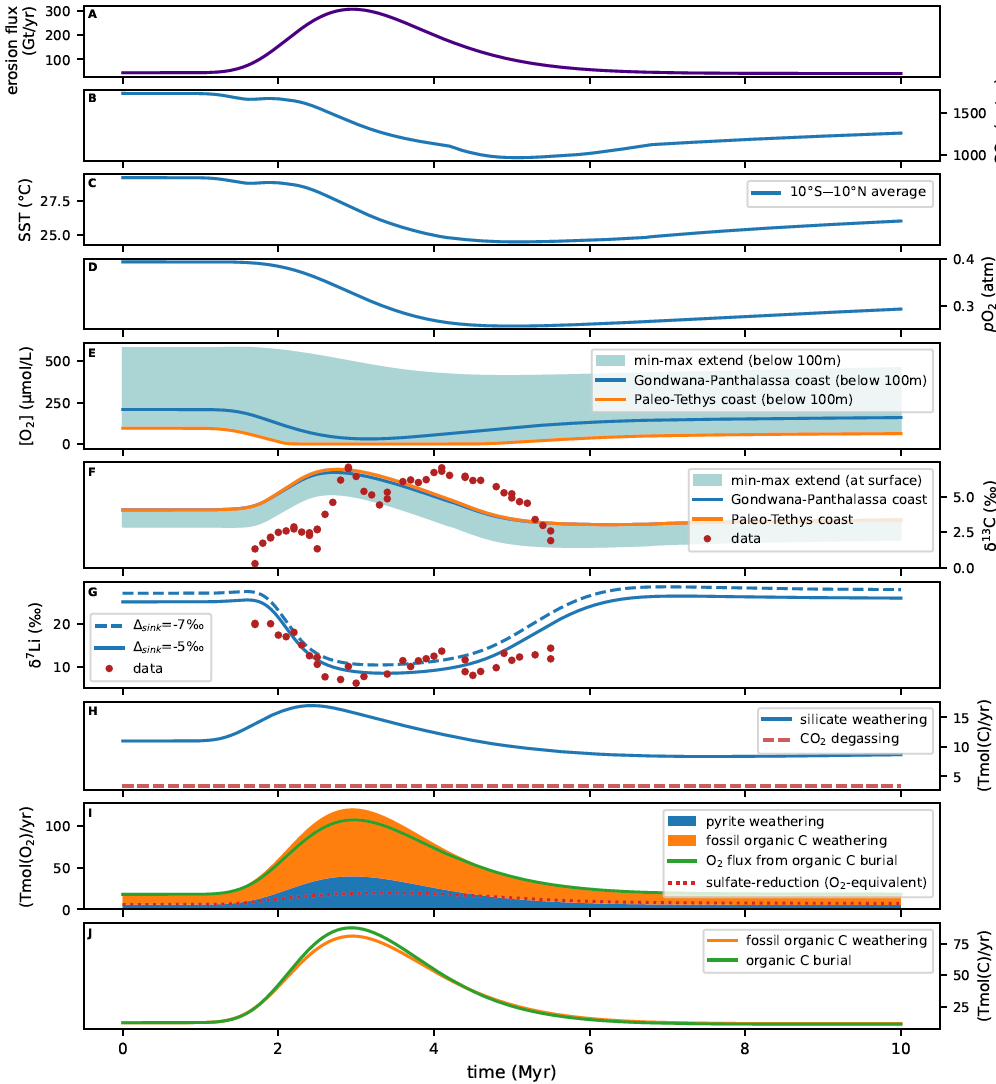


Supplementary Figure S11. Same than main text Fig. 4 for the GEOCLIM simulation with 75% of “explicit” reverse weathering (*f_rw_*=0.75).


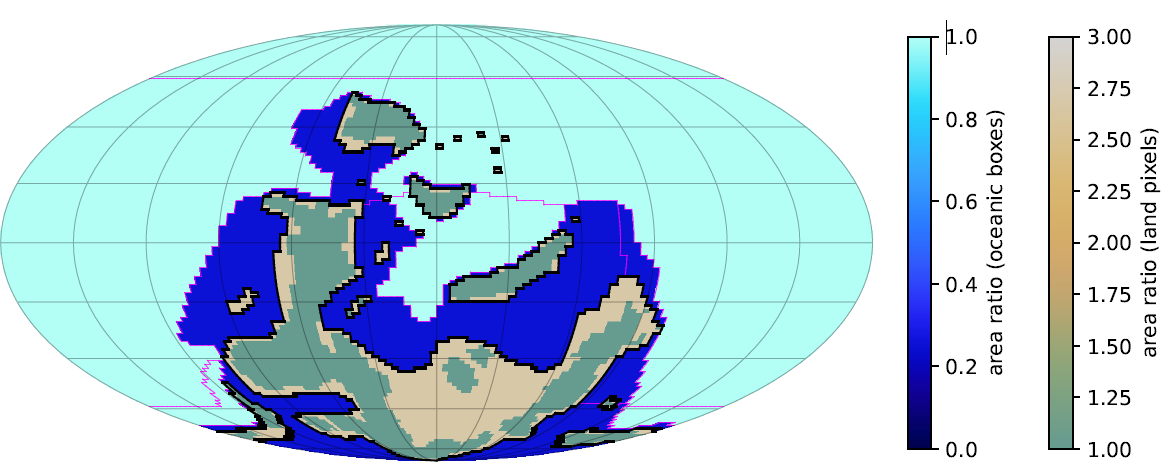


Supplementary Figure S12. Map showing the area ratio between the GEOCLIM simulation with reduced extent of inundated shelves and the main text simulation. This area ratio concerns the oceanic boxes shallower than 250m and the land pixels lower than 250m.


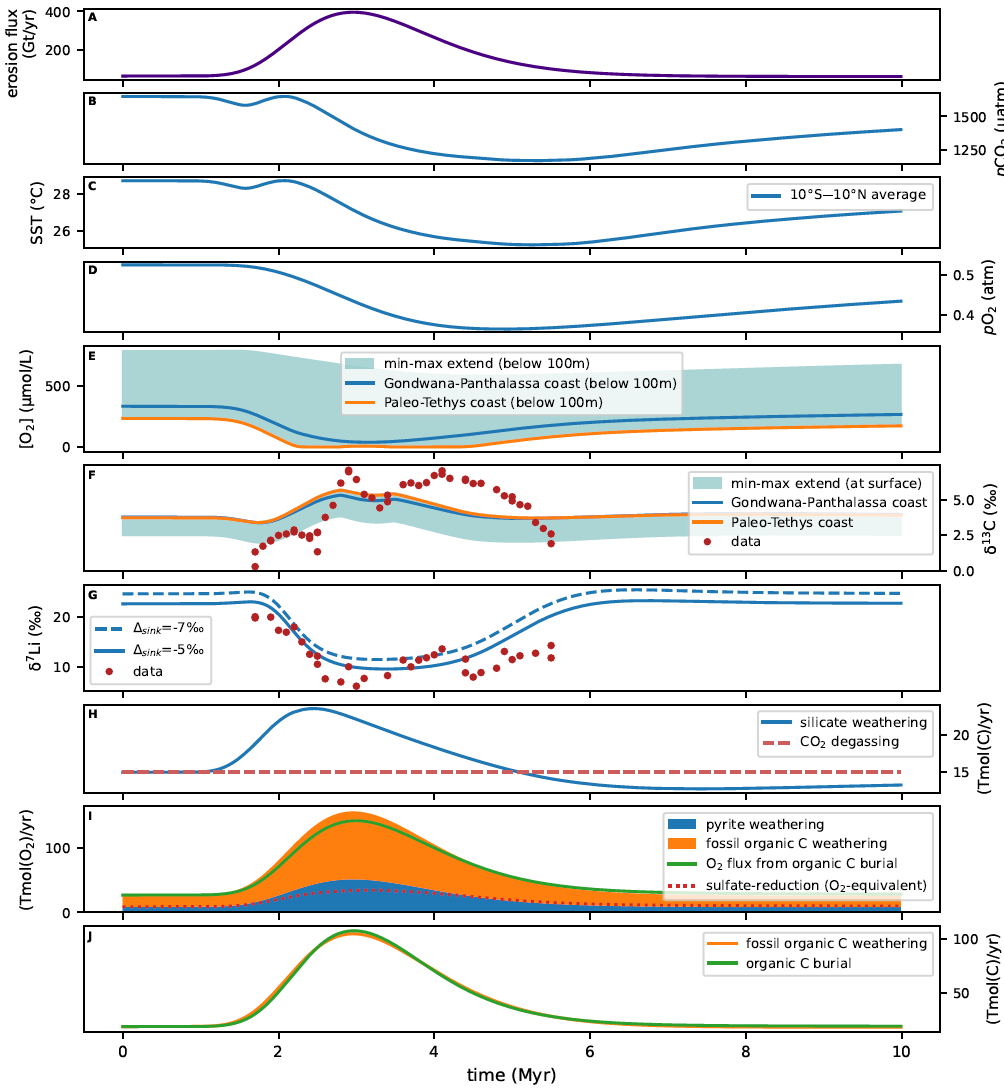


**Supplementary Figure S13**. Same than main text Fig. 4 for the GEOCLIM simulation with reduced extent of inundated shelves (and increased extent of lowlands).

**Supplementary Table S1: Lithium concentration and δ^7^Li data with associated geochemical data from the Pahranagat Range.**

| Sample ID | δ^13^C (VPDB, ‰) | δ^18^O (VPDB, ‰) | δ^7^Li (‰) | Li_carb_ (ppm) | Height (m) | Al (ppm) | Ca  (%) | Mg (%) | Mn (ppm) | Sr (ppm) | Rb (ppm) | Th (ppm) | U (ppm) |
| --- | --- | --- | --- | --- | --- | --- | --- | --- | --- | --- | --- | --- | --- |
| PR-11 | 0.31 | -4.54 | 16.05 | 0.11 | 11 | 23.50 | 36.48 | 0.24 | 21.02 | 428.40 | 0.15 | 0.01 | 0.14 |
| PR-15.5 | 1.34 | -4.94 | 15.80 | 0.13 | 15.5 | 10.77 | 37.41 | 0.40 | 15.62 | 293.53 | 0.20 | 0.00 | 0.15 |
| PR-19 | 1.74 | -3.90 |  | 0.26 | 19 | 8.24 | 36.05 | 0.28 | 14.84 | 322.63 | 0.12 | 0.00 | 0.27 |
| PR-24 | 2.11 | -4.97 | 15.97 | 0.21 | 24 | 7.49 | 36.34 | 0.30 | 15.24 | 339.98 | 0.05 | 0.00 | 0.21 |
| PR-28 | 2.16 | -5.39 |  | 0.16 | 28 | 7.39 | 36.02 | 0.22 | 16.13 | 361.58 | 0.07 | 0.00 | 0.53 |
| PR-32 | 2.50 | -4.31 | 13.33 | 0.14 | 32 | 8.24 | 36.91 | 0.32 | 17.53 | 329.22 | 0.19 | 0.00 | 0.29 |
| PR-36.5 | 2.60 | -4.21 | 12.95 | 0.14 | 36.5 | 7.23 | 39.55 | 0.32 | 19.58 | 421.64 | 0.17 | 0.00 | 0.30 |
| PR-41.1 | 2.88 | -5.01 | 13.98 | 0.09 | 41.1 | 4.41 | 37.93 | 0.30 | 41.84 | 730.88 | 0.07 | 0.00 | 0.29 |
| PR-46 | 2.74 | -5.28 | 12.34 | 0.10 | 46 | 28.15 | 36.46 | 0.21 | 17.96 | 345.53 | 0.21 | 0.00 | 0.47 |
| PR-50 | 2.52 | -5.78 | 11.01 | 0.10 | 50 | 6.23 | 36.54 | 0.25 | 18.95 | 335.16 | 0.09 | 0.00 | 0.76 |
| PR-54 | 2.29 | -8.40 | 8.53 | 0.08 | 54 | 29.29 | 36.28 | 0.27 | 23.12 | 334.76 | 0.27 | 0.00 | 0.56 |
| PR-58 | 2.47 | -8.56 |  | 0.16 | 58 | 39.53 | 28.73 | 0.25 | 27.74 | 402.21 | 0.18 | 0.00 | 0.25 |
| PR-62.5 | 2.69 | -5.43 | 8.16 | 0.19 | 62.5 | 10.75 | 37.64 | 0.26 | 33.62 | 606.96 | 0.07 | 0.00 | 0.28 |
| PR-64 | 1.35 | -5.72 |  | 0.18 | 64 | 20.81 | 32.14 | 0.26 | 59.58 | 542.53 | 0.15 | 0.00 | 0.26 |
| PR-66 | 2.72 | -5.97 | 6.53 | 0.15 | 66 | 11.38 | 37.46 | 0.26 | 67.37 | 250.29 | 0.06 | 0.00 | 0.20 |
| PR-70 | 3.76 | -4.97 | 3.62 | 0.14 | 70 | 21.56 | 35.00 | 0.25 | 34.57 | 445.37 | 0.27 | 0.00 | 0.12 |
| PR-75.3 | 4.60 | -3.75 |  | 0.27 | 75.3 | 36.16 | 31.24 | 0.28 | 29.59 | 567.37 | 0.26 | 0.02 | 0.13 |
| PR-80 | 6.16 | -3.41 | 2.99 | 0.22 | 80 | 74.54 | 33.16 | 0.29 | 46.03 | 674.64 | 0.63 | 0.01 | 0.11 |
| PR-85.5 | 6.95 | -3.57 |  | 0.23 | 85.5 | 79.38 | 33.33 | 0.37 | 34.24 | 721.96 | 0.75 | 0.08 | 0.05 |
| PR-89 | 7.06 | -3.10 | 6.04 | 0.26 | 89 | 70.58 | 33.87 | 0.55 | 37.90 | 779.48 | 0.78 | 0.19 | 0.05 |
| PR-95 | 6.41 | -3.98 | 2.15 | 0.14 | 95 | 63.50 | 33.99 | 0.34 | 35.12 | 621.84 | 0.76 | 0.19 | 0.20 |
| PR-102 | 5.37 | -3.41 | 3.69 | 0.27 | 102 | 136.70 | 28.06 | 0.61 | 35.62 | 723.52 | 0.89 | 0.25 | 0.11 |
| PR-105 | 5.12 | -3.00 |  |  | 105 | 289.75 | 24.09 | 0.58 | 36.42 | 668.95 | 1.67 | 0.34 | 0.13 |
| PR-113.1 | 4.42 | -5.80 |  |  | 113.1 | 158.62 | 24.52 | 1.09 | 29.87 | 549.07 | 1.04 | 0.14 | 0.13 |
| PR-119.4 | 4.85 | -3.68 | 4.92 | 0.29 | 119.4 | 46.46 | 31.90 | 0.34 | 27.81 | 523.24 | 0.44 | 0.13 | 0.14 |
| PR-122.1 | 5.31 | -3.69 | 4.27 | 0.25 | 122.1 | 53.15 | 31.77 | 0.27 | 27.68 | 806.18 | 0.63 | 0.12 | 0.10 |
| PR-130 | 6.05 | -3.49 | 7.38 | 0.23 | 130 | 46.25 | 33.72 | 0.36 | 26.46 | 740.95 | 0.43 | 0.08 | 0.07 |
| PR-136 | 6.17 | -3.25 | 6.02 | 0.34 | 136 | 61.41 | 33.16 | 1.01 | 19.30 | 750.56 | 0.33 | 0.02 | 0.07 |
| PR-144 | 5.99 | -3.63 | 7.35 | 0.18 | 144 | 52.44 | 30.64 | 0.76 | 22.12 | 670.97 | 0.41 | 0.08 | 0.05 |
| PR-149.9 | 6.19 | -3.04 | 7.84 | 0.21 | 149.9 | 31.70 | 34.27 | 0.37 | 24.86 | 703.22 | 0.45 | 0.04 | 0.09 |
| PR-156.8 | 6.69 | -4.25 | 8.43 | 0.12 | 156.8 | 34.22 | 33.70 | 0.33 | 15.89 | 716.55 | 0.17 | 0.02 | 0.13 |
| PR-162 | 7.02 | -3.21 |  | 0.28 | 162 | 27.05 | 27.90 | 0.40 | 19.81 | 791.89 | 0.17 | 0.00 | 0.09 |
| PR-166 | 6.78 | -3.70 | 9.60 | 0.27 | 166 | 23.56 | 29.51 | 0.28 | 24.81 | 794.09 | 0.42 | 0.06 | 0.05 |
| PR-172 | 6.49 | -3.44 |  | 0.26 | 172 | 69.82 | 31.24 | 0.36 | 26.39 | 706.97 | 0.62 | 0.15 | 0.12 |
| PR-181 | 6.43 | -3.30 | 7.57 | 0.30 | 181 | 53.95 | 28.23 | 0.39 | 28.01 | 676.43 | 0.53 | 0.21 | 0.05 |
| PR-185 | 6.35 | -3.54 | 4.83 | 0.25 | 185 | 32.80 | 30.11 | 0.27 | 23.08 | 787.11 | 0.30 | 0.12 | 0.09 |
| PR-192 | 6.13 | -3.45 | 3.97 | 0.24 | 192 | 51.23 | 35.49 | 0.29 | 28.94 | 745.27 | 1.01 | 0.20 | 0.09 |
| PR-198 | 6.14 | -2.95 | 4.87 | 0.28 | 198 | 72.92 | 34.65 | 0.59 | 22.90 | 624.83 | 0.72 | 0.16 | 0.05 |
| PR-207 | 5.70 | -3.34 | 5.74 | 0.20 | 207 | 44.77 | 32.92 | 0.29 | 23.39 | 698.95 | 0.60 | 0.10 | 0.07 |
| PR-213 | 5.27 | -3.11 | 9.09 | 0.22 | 213 | 39.31 | 35.22 | 0.57 | 31.11 | 546.42 | 0.57 | 0.21 | 0.09 |
| PR-218 | 5.20 | -3.17 | 8.33 | 0.19 | 218 | 81.12 | 29.44 | 0.50 | 26.93 | 569.44 | 0.63 | 0.23 | 0.12 |
| PR-221 | 4.91 | -3.67 | 7.47 | 0.27 | 221 | 61.91 | 34.76 | 0.30 | 29.62 | 552.25 | 0.71 | 0.18 | 0.11 |
| PR-227 | 4.65 | -3.90 | 8.24 | 0.33 | 227 | 54.69 | 31.79 | 0.27 | 21.94 | 549.78 | 0.56 | 0.13 | 0.12 |
| PR-231 | 4.54 | -3.16 |  | 0.32 | 231 | 33.14 | 32.80 | 0.38 | 21.84 | 606.39 | 0.26 | 0.04 | 0.14 |
| PR-239 | 3.40 | -3.62 | 8.74 | 0.27 | 239 | 43.33 | 34.74 | 0.33 | 30.14 | 517.55 | 0.37 | 0.08 | 0.20 |
| PR-244 | 2.98 | -4.39 | 7.25 | 0.30 | 244 | 48.85 | 33.14 | 0.31 | 32.55 | 518.06 | 0.60 | 0.15 | 0.22 |
| PR-250 | 2.60 | -3.39 | 7.81 | 0.27 | 250 | 25.59 | 37.44 | 0.31 | 31.68 | 391.71 | 0.14 | 0.02 | 0.18 |
| PR-252 | 1.92 | -4.26 | 10.29 | 0.26 | 252 | 31.96 | 30.30 | 0.60 | 32.89 | 291.30 | 0.25 | -0.01 | 0.11 |

**Supplementary Table S2: Lithium concentration and δ^7^Li data with associated geochemical data from the Sacagawea Peak.**

| Sample ID | δ^13^C (VPDB, ‰) | δ^18^O (VPDB, ‰) | δ^7^Li (‰) | Li_carb_ (ppm) | Height (m) | Al (ppm) | Ca (%) | Mg (%) | Mn (ppm) | Sr (ppm) | Rb (ppm) | Th (ppm) | U (ppm) |
| --- | --- | --- | --- | --- | --- | --- | --- | --- | --- | --- | --- | --- | --- |
| SP-4.5 | -0.80 | -8.38 | 14.46 | 0.42 | 4.5 | 30.02 | 18.14 | 3.77 | 71.46 | 25.38 | 0.16 | 0.03 | 0.05 |
| SP-9 | -0.06 | -4.99 |  |  | 9 | 206.11 | 12.53 | 6.94 | 159.40 | 34.17 | 0.22 | 0.21 | 0.11 |
| SP-12 | 0.14 | -3.40 | 15.44 | 1.02 | 12 |  |  |  |  |  |  |  |  |
| SP-28.5 | -2.50 | -12.32 | 14.23 |  | 28.5 |  |  |  |  |  |  |  |  |
| SP-30.4 | -1.80 | -12.79 | 12.98 | 0.26 | 30.4 |  |  |  |  |  |  |  |  |
| SP-36 | 0.29 | -11.17 | 12.35 | 0.32 | 36 | 6.71 | 27.35 | 0.12 | 17.49 | 100.03 | 0.06 | 0.00 | 0.76 |
| SP-90 | 6.31 | -3.97 | 8.76 | 1.13 | 90 | 80.26 | 33.45 | 0.56 | 73.01 | 639.10 | 0.22 | 0.40 | 0.11 |
| SP-110 | 6.08 | -4.44 | 7.51 | 0.74 | 110 |  |  |  |  |  |  |  |  |
| SP-120 | 5.94 | -4.66 | 6.66 | 1.00 | 120 | 91.39 | 27.75 | 0.29 | 120.06 | 443.32 | 0.30 | 1.09 | 0.16 |
| SP-123 | 6.26 | -4.02 | 9.08 | 0.98 | 123 |  |  |  |  |  |  |  |  |
| SP-130.1 | 5.92 | -3.50 | 10.21 | 1.16 | 130.1 | 69.89 | 32.23 | 0.42 | 63.16 | 1018.67 | 0.25 | 0.40 | 0.10 |
| SP-137 | 5.78 | -3.23 | 8.94 | 0.90 | 137 |  |  |  |  |  |  |  |  |
| SP-139.8 | 5.13 | -4.86 | 8.24 | 0.68 | 139.8 | 27.74 | 32.55 | 0.26 | 174.68 | 629.79 | 0.13 | 0.28 | 0.07 |
| SP-148.5 | 5.95 | -3.19 |  |  | 148.5 | 256.80 | 30.10 | 0.67 | 75.14 | 726.74 | 0.39 | 0.46 | 0.10 |
| SP-149.5 | 2.92 | -8.00 |  |  | 149.5 |  |  |  |  |  |  |  |  |
| SP-153 | 5.64 | -4.99 |  |  | 153 | 349.55 | 27.26 | 0.60 | 134.27 | 864.39 | 0.43 | 1.38 | 0.09 |
| SP-160.4 | 2.77 | -4.34 | 5.13 | 0.12 | 160.4 |  |  |  |  |  |  |  |  |
| SP-169.9 | 7.19 | -3.53 |  | 0.88 | 169.9 | 20.56 | 34.47 | 0.38 | 40.93 | 1083.27 | 0.13 | 0.21 | 0.13 |
| SP-170 | 6.86 | -3.50 | 5.36 | 0.58 | 170 |  |  |  |  |  |  |  |  |
| SP-190.2 | 6.99 | -4.32 | 4.30 | 1.10 | 190.2 | 92.87 | 32.46 | 0.30 | 98.02 | 885.07 | 0.21 | 0.65 | 0.06 |
| SP-200.9 | 6.16 | -5.53 | 6.66 | 0.46 | 200.9 | 37.01 | 18.39 | 0.19 | 38.89 | 412.99 | 0.18 | 0.69 | 0.07 |
| SP-221.5 | 5.18 | -3.48 | 6.68 | 0.78 | 221.5 |  |  |  |  |  |  |  |  |
| SP-230.3 | 6.57 | -4.13 |  |  | 230.3 | 16.80 | 22.02 | 0.26 | 22.91 | 565.45 | 0.05 | 0.01 | 0.74 |
| SP-241 | 6.76 | -3.59 | 7.97 | 0.94 | 241 |  |  |  |  |  |  |  |  |
| SP-250 | 5.60 | -4.54 | 7.85 | 0.77 | 250 | 27.16 | 34.05 | 0.38 | 47.98 | 617.60 | 0.16 | 0.28 | 0.12 |
| SP-271 | 3.48 | -6.09 | 5.60 | 0.68 | 271 | 79.90 | 34.48 | 0.37 | 27.92 | 264.08 | 0.21 | 0.14 | 0.57 |
| SP-275.5 | 2.05 | -6.47 | 3.14 | 0.20 | 275.5 |  |  |  |  |  |  |  |  |
| SP-281 | 2.93 | -5.20 | 3.21 | 0.23 | 281 |  |  |  |  |  |  |  |  |
| SP-286 | 2.60 | -6.56 |  | 0.21 | 286 | 5.29 | 27.55 | 0.15 | 11.63 | 82.58 | 0.09 | 0.00 | 0.39 |
| SP-290.6 | 2.75 | -7.47 | 2.65 | 0.13 | 290.6 |  |  |  |  |  |  |  |  |
| SP-291 | 2.87 | -6.25 | 1.73 | 0.51 | 291 | 61.03 | 28.39 | 2.22 | 13.99 | 86.56 | 0.06 | 0.10 | 0.42 |
| SP-296 | 2.53 | -7.88 | 2.05 | 0.07 | 296 |  |  |  |  |  |  |  |  |
| SP-300 | 2.36 | -5.70 | 0.51 | 0.58 | 300 | 10.81 | 36.79 | 0.21 | 13.50 | 185.31 | 0.07 | 0.02 | 0.72 |
| SP-328.8 | 2.09 | -3.87 | 6.07 | 0.99 | 328.8 | 19.70 | 36.17 | 0.27 | 11.18 | 217.88 | 0.08 | 0.03 | 0.82 |
| SP-330 | -0.08 | -12.03 | 5.17 | 0.26 | 330 |  |  |  |  |  |  |  |  |
| SP-350 | 1.70 | -5.89 | 5.18 | 1.02 | 350 | 15.41 | 35.51 | 0.25 | 14.23 | 242.82 | 0.07 | 0.02 | 1.19 |
| SP-355.5 | 1.24 | -6.09 | 3.29 | 0.78 | 355.5 | 25.29 | 36.52 | 0.22 | 11.59 | 220.42 | 0.09 | 0.04 | 1.77 |
| SP-370 | -0.34 | -11.43 |  |  | 370 |  |  |  |  |  |  |  |  |
| SP-390.3 | 2.04 | -5.79 | 2.20 | 0.67 | 390.3 | 9.94 | 35.06 | 0.20 | 13.05 | 149.46 | 0.08 | 0.04 | 1.63 |
| SP-400 | 2.53 | -6.77 | 13.06 | 0.68 | 400 | 126.23 | 16.63 | 8.36 | 23.11 | 42.38 | 0.05 | 0.01 | 1.50 |
| SP-407 | 0.17 | -11.52 |  |  | 407 |  |  |  |  |  |  |  |  |
| SP-411.2 | 1.75 | -12.09 | 12.07 | 0.11 | 411.2 | 14.91 | 34.83 | 0.29 | 37.28 | 61.03 | 0.09 | 0.03 | 0.03 |
| SP-415.4 | 1.63 | -12.63 |  |  | 415.4 |  |  |  |  |  |  |  |  |
| SP-424.2 | 3.08 | -5.45 | 11.74 | 0.58 | 424.2 | 35.86 | 13.57 | 6.75 | 33.13 | 27.67 | 0.05 | 0.01 | 1.70 |
| SP-425.4 | 2.59 | -1.79 | 15.29 | 0.66 | 425.4 | 78.43 | 12.03 | 6.83 | 22.46 | 17.34 | 0.07 | 0.01 | 1.04 |
| SP-428.4 | 2.65 | -10.02 |  |  | 428.4 |  |  |  |  |  |  |  |  |
| SP-433.2 | 2.91 | 0.05 | 19.41 | 0.42 | 433.2 | 71.16 | 11.93 | 5.50 | 19.52 | 26.60 | 0.10 | 0.00 | 0.42 |
| SP-451.8 | 1.95 | -6.00 |  |  | 451.8 |  |  |  |  |  |  |  |  |
| SP-458.7 | 2.91 | -3.40 |  |  | 458.7 | 274.32 | 16.90 | 6.00 | 40.44 | 113.72 | 0.36 | 0.69 | 0.19 |
| SP-474.5 | 2.66 | -6.96 |  |  | 474.5 |  |  |  |  |  |  |  |  |
| SP-480.3 | 1.65 | -3.91 | 18.04 | 0.60 | 480.3 | 79.73 | 14.72 | 5.83 | 39.88 | 19.66 | 0.05 | 0.01 | 0.44 |
| SP-482.3 | 1.91 | -3.14 | 18.18 | 0.66 | 482.3 |  |  |  |  |  |  |  |  |
| SP-488 | 2.39 | -0.11 | 20.41 | 0.39 | 488 | 40.04 | 6.23 | 3.10 | 16.16 | 9.54 | 0.05 | 0.03 | 0.27 |

**Supplementary Table S3: Modified COPSE forcings and paramteters. Compare to the standard COPSE reloaded configuration (16), here, only minor modifcations were made to achieve a high pCO_2_ pre-TICE steady state. Unless stated below, all the other COPSE forcings and parameters are the same as those of COPSE_reloaded_version (16) at 351 Ma.**

| **Process/Forcing** | **Label** | **Value** | **Units** | **References/Notes** |
| --- | --- | --- | --- | --- |
| ***Model Forcing (at ~351 Ma)*** |  |  |  |  |
| Metamorphic and volcanic degassing | D | 1.60 | - | to achieve the 351 Ma steady state |
| Tectonic uplift | U | 0.80 | - | to achieve the 351 Ma steady state |
| Mass of terrestrial biosphere | VEG | 0.90 | - | ref.16 |
| Normalised granite area | a_gran_ | 1.40 | - | ref.16 |
| Normalised basalt area | a_bas_ | 1.0 | - | ref.16 |
|  |  |  |  |  |
| ***Carbon cycle*** |  |  |  |  |
| Carbon isotope of sedimentary carbonate carbon | δ^13^C_C | 0.00 | ‰ | to get δ^13^C_DIC = 2‰ |
| Carbon isotope of sedimentary organic carbon | δ^13^C_G | -24.0 | ‰ | to get δ^13^C_DIC = 2‰ |

**Supplementary References**

1. Maharjan D, Jiang G, Peng Y *et al.* Sulfur isotope change across the Early Mississippian K–O (Kinderhookian–Osagean) δ13C excursion. *Earth and Planetary Science Letters*. 2018; **494**: 202–215. doi: 10.1016/j.epsl.2018.04.043

2. Romaniello SJ, Elrick M, Cheng K. Early Mississippian ocean anoxia triggered organic carbon burial and late Paleozoic cooling: Evidence from uranium isotopes recorded in marine limestone. *Geology*. 2020; **48**(4): 363–367. doi: 10.1130/g46950.1

3. Wei G-Y, Zhang F, Yin Y-S *et al.* A 13 million-year record of Li isotope compositions in island carbonates: Constraints on bulk inorganic carbonate as a global seawater Li isotope archive. *Geochimica et Cosmochimica Acta*. 2023; **344**: 59–72. doi: 10.1016/j.gca.2023.01.013

4. Pogge von Strandmann PAE, Jenkyns HC, Woodfine RG. Lithium isotope evidence for enhanced weathering during Oceanic Anoxic Event 2. *Nature Geoscience*. 2013; **6**(8): 668–672. doi: 10.1038/ngeo1875

5. Pogge von Strandmann PAE, Jones MT, West AJ *et al.* Lithium isotope evidence for enhanced weathering and erosion during the Paleocene-Eocene Thermal Maximum. *Science Advances*. 2021; **7**(42): eabh4224.

6. Maffre P, Goddéris Y, Le Hir G *et al.* GEOCLIM7, an Earth System Model for multi-million years evolution of the geochemical cycles and climate. *Geosci Model Dev Discuss*. 2024; **2024**: 1–66. doi: 10.5194/gmd-2024-220

7. Caves Rugenstein JK, Ibarra DE, von Blanckenburg F. Neogene cooling driven by land surface reactivity rather than increased weathering fluxes. *Nature*. 2019; **571**(7763): 99–102. doi: 10.1038/s41586-019-1332-y

8. Gaillardet J, Dupré B, Louvat P *et al.* Global silicate weathering and CO2 consumption rates deduced from the chemistry of large rivers. *Chemical Geology*. 1999; **159**(1): 3–30. doi: <https://doi.org/10.1016/S0009-2541(99)00031-5>

9. Misra S, Froelich PN. Lithium isotope history of Cenozoic seawater: changes in silicate weathering and reverse weathering. *Science*. 2012; **335**(6070): 818–823. doi: 10.1126/science.1214697

10. Jacob RL. Low frequency variability in a simulated atmosphere-ocean system *PhD.* The University of Wisconsin-Madison, 1997.

11. Scotese CR, Wright N. PALEOMAP paleodigital elevation models (PaleoDEMS) for the Phanerozoic. *Paleomap Proj*. 2018: 1–26.

12. Gough D. Solar interior structure and luminosity variations. *Solar Physics*. 1981; **74**(1): 21–34.

13. Goddéris Y, Joachimski MM. Global change in the Late Devonian: modelling the Frasnian–Famennian short-term carbon isotope excursions. *Palaeogeography, Palaeoclimatology, Palaeoecology*. 2004; **202**(3-4): 309–329. doi: 10.1016/s0031-0182(03)00641-2

14. Pohl A, Donnadieu Y, Le Hir G *et al.* Effect of the Ordovician paleogeography on the (in)stability of the climate. *Clim Past*. 2014; **10**(6): 2053–2066. doi: 10.5194/cp-10-2053-2014

15. Chen B, Chen J, Qie W *et al.* Was climatic cooling during the earliest Carboniferous driven by expansion of seed plants? *Earth and Planetary Science Letters*. 2021; **565**: 116953. doi: <https://doi.org/10.1016/j.epsl.2021.116953>

16. Kalderon-Asael B, Katchinoff JAR, Planavsky NJ *et al.* A lithium-isotope perspective on the evolution of carbon and silicon cycles. *Nature*. 2021; **595**(7867): 394–398. doi: 10.1038/s41586-021-03612-1

17. Misra S, Froelich, P. N. Lithium isotope History of Cenozoic Seawater: Changes in Silicate Weathering and Reverse Weathering. *Science*. 2012; **335**: 818–823.

18. Marcilly CM, Torsvik TH, Domeier M *et al.* New paleogeographic and degassing parameters for long-term carbon cycle models. *Gondwana Research*. 2021; **97**: 176–203. doi: 10.1016/j.gr.2021.05.016

19. Marcilly CM, Torsvik TH, Jones MT. Late Paleozoic climate transition from a long-term carbon cycle modeling perspective. *Global and Planetary Change*. 2025; **253**. doi: 10.1016/j.gloplacha.2025.104984

20. Lenton TM, Daines SJ, Mills BJW. COPSE reloaded: An improved model of biogeochemical cycling over Phanerozoic time. *Earth-Science Reviews*. 2018; **178**: 1–28. doi: 10.1016/j.earscirev.2017.12.004

21. Pogge von Strandmann PAE, Desrochers A, Murphy MJ *et al.* Global climate stabilisation by chemical weathering during the Hirnantian glaciation. *Geochemical Perspectives Letters*. 2017; **3**: 230–237. doi: 10.7185/geochemlet.1726

22. Pogge von Strandmann PAE, Kasemann SA, Wimpenny JB. Lithium and Lithium Isotopes in Earth’s Surface Cycles. *Elements*. 2020; **16**(4): 253–258. doi: 10.2138/gselements.16.4.253

23. Pourmand A, Dauphas N, Ireland TJ. A novel extraction chromatography and MC-ICP-MS technique for rapid analysis of REE, Sc and Y: Revising CI-chondrite and Post-Archean Australian Shale (PAAS) abundances. *Chemical Geology*. 2012; **291**: 38–54. doi: 10.1016/j.chemgeo.2011.08.011
